# Integrative Analysis of Transcriptome and Metabolome to Illuminate the Protective Effects of Didymin against Acute Hepatic Injury

**DOI:** 10.1155/2023/6051946

**Published:** 2023-01-12

**Authors:** Lijun Pang, Yuhua Xiong, Zhongwen Feng, Cuiyu Li, Bin Fang, Quanfang Huang, Xing Lin

**Affiliations:** ^1^Guangxi Medical University, Nanning 530021, China; ^2^The First Affiliated Hospital of Guangxi University of Chinese Medicine, Nanning 530023, China

## Abstract

Based on the multiomics analysis, this study is aimed at investigating the underlying mechanism of didymin against acute liver injury (ALI). The mice were administrated with didymin for 2 weeks, followed by injection with lipopolysaccharide (LPS) plus D-galactosamine (D-Gal) to induce ALI. The pathological examination revealed that didymin significantly ameliorated LPS/D-Gal-induced hepatic damage. Also, it markedly reduced proinflammatory cytokines release by inhibiting the TLR4/NF-*κ*B pathway activation, alleviating inflammatory injury. A transcriptome analysis proved 2680 differently expressed genes (DEGs) between the model and didymin groups and suggested that the PI3K/Akt and metabolic pathways might be the most relevant targets. Meanwhile, the metabolome analysis revealed 67 differently expressed metabolites (DEMs) between the didymin and model groups that were mainly clustered into the glycerophospholipid metabolism, which was consistent with the transcriptome study. Importantly, a comprehensive analysis of both the omics indicated a strong correlation between the DEGs and DEMs, and an in-depth study demonstrated that didymin alleviated metabolic disorder and hepatocyte injury likely by inhibiting the glycerophospholipid metabolism pathway through the regulation of PLA2G4B, LPCAT3, and CEPT1 expression. In conclusion, this study demonstrates that didymin can ameliorate LPS/D-Gal-induced ALI by inhibiting the glycerophospholipid metabolism and PI3K/Akt and TLR4/NF-*κ*B pathways.

## 1. Introduction

Acute liver injury can be induced by various risk factors, such as alcohol, lipopolysaccharide (LPS), ionizing radiation, chemotherapy, and acetaminophen. LPS-induced liver injury is defined as a class of syndrome with liver inflammatory responses [1]. As an important constituent of Gram-negative bacteria cell walls, LPS has strong toxicity and often contributes to multiorgan failure, especially in liver injury [2, 3]. Moreover, D-galactosamine (D-Gal), as a sensitizer for LPS, can enhance the hepatotoxicity of LPS in the liver [4]. Therefore, LPS/D-Gal-induced ALI has been widely used as a model to evaluate the efficiency of hepatoprotective agents [5].

Recently, multiomics analysis has been considered a promising strategy to elucidate the complicated pathogenesis of multiple diseases [6, 7]. Transcriptomics that can detect the complete set of RNA transcripts is a useful tool for studying the pathogenesis of various diseases and drug discovery because of its advantage in providing insight into the potential biomarkers and mechanisms as a whole [8]. Metabolomics can provide the global metabolic profile and expose the relationship between abnormal metabolism and pathological mechanisms to some extent. In our recent work, we have found that the comprehensive analysis of transcriptomics and metabolomics was a feasible measure to elucidate the complicated mechanism of helenalin on hepatic fibrosis [9]. Increasing evidence has indicated that the integrative analysis of transcriptomics and metabolomics might be an efficient measure to predict the targets of medicines [10, 11].

Didymin, also known as isosakuranetin-7-O-rutinoside, is a common flavonoid found in many kinds of herbs, such as *Origanum vulgare* L. [12]. Didymin has been proven to be a potent antioxidant and possesses therapeutic effects on several types of tumors, such as lung cancer, breast cancer, and brain tumor [13]. In our previous study, we found that didymin could ameliorate dexamethasone-induced nonalcoholic fatty liver disease in C57BL/6J mice [14], suggesting that didymin might have the potential for the treatment of liver steatosis. Next, we wanted to know whether didymin could be used as a remedy to treat various liver diseases and planned to develop it as a hepatic protectant in the future, which meant the effects of didymin on multiple experimental models of liver diseases should be assessed. As a part of our plans, the aim of the current study was to evaluate the hepatoprotective effect of didymin by using the LPS/D-Gal-induced liver injury model in mice. Also, the possible targets would be predicted by the transcriptomics and further verified by the metabolomics analysis and the multiple relevant examinations.

## 2. Materials and Methods

### 2.1. Animals and Treatments

Male C57BL/6J mice (20 ± 2 g, 6–8 weeks old, SPF) were obtained from Changsha Tianqin Biotechnology Co., LTD (Quality certificate number: SYXK Xiang 2014–0011, Changsha, China). The animal study was approved by the Experimental Animal Center of Guangxi Medical University (Ethics Committee number: 201901053, Guangxi, China).

**Figure 1:**
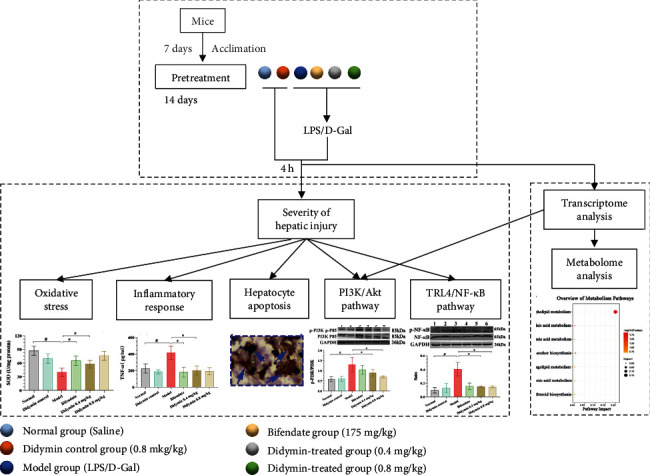
Design of this research. (a) The schedule of raising mice. (b) Animal experiments were conducted. (c) Transcriptomics and metabolomics analysis.

The schedule of animal experiments is shown in Figure 1. After one week of acclimatizing, all mice were randomly divided into six groups (*n* = 10): normal control group, didymin control group (0.8 mg/kg didymin), model group, bifendate-treated group (positive control; 175 mg/kg bifendate) [7], and didymin-treated groups (0.4 or 0.8 mg/kg didymin). All groups received the corresponding medicine once a day for two weeks. After that, except for the normal and didymin control groups, the mice in other groups were injected with LPS (50 *μ*g/kg) and D-Gal (800 mg/kg) intraperitoneally to induce ALI [15]. After 4 h of the treatment with LPS/D-Gal, all mice were anesthetized by intraperitoneal injection of 3% sodium pentobarbital (1.0 ml/kg). Blood and liver samples were collected immediately.

### 2.2. Histological Examination of Liver Tissues

Fresh liver tissues were fixed in 4% paraformaldehyde, embedded in paraffin, and sliced into sections [16]. Liver histology was assessed using hematoxylin and eosin (H&E) staining. Liver histopathological changes were evaluated with an optical microscope (Nikon, Tokyo, Japan).

### 2.3. Biochemical Analyses

Activities of serum alanine aminotransferase (ALT), aspartate aminotransferase (AST), total protein (TP), albumin (ALB), globulin (GLB), and A/G (ALB/GLB) were determined using commercial kits by an Automatic Blood Biochemical Analyzer (7600-120, Hitachi High-Tech, Corp, Tokyo, Japan).

### 2.4. Oxidative Stress and Lipid Peroxidation Levels

The enzymes in the supernatants, including glutathione reductase (GSH-Rd), superoxide dismutase (SOD), myeloperoxidase (MPO), malondialdehyde (MDA), and inducible nitric oxide synthase (iNOS), were detected using the commercially available kits (Jiancheng Bioengineering Institute of Nanjing, Nanjing, China) according to the manufacturer's protocols. Hepatic reactive oxygen species (ROS) were detected using an ELISA kit (FANKEWEI, Shanghai, China).

### 2.5. Determination of Inflammatory Cytokines in Serum

Inflammatory cytokines in serum, including interleukin-6 (IL-6), interleukin-1 beta (IL-1*β*), and tumor necrosis factor-*α* (TNF-*α*), were determined using the commercial ELISA kits (Elabscience Biotechnology Co. Ltd., Wuhan, Hubei, China) according to the manufacturer's instructions.

### 2.6. TUNEL Staining

Hepatocyte apoptosis was determined by the TUNEL method using the In Situ Apoptosis Detection Kit. Briefly, the liver tissue sections were dewaxed and treated with proteinase K, followed by incubation with TUNEL detection buffer in the dark for 1 h at 37°C according to the manufacturer's instructions.

### 2.7. Transcriptome Analysis

The transcriptome analysis was conducted as described in our previous study [9]. Briefly, total RNA from liver tissues was extracted with Trizol reagent (Thermo Fisher Scientific, Lot. 15596018). RNA sequences were analyzed by Shanghai Jiayin Biotechnology Co., Ltd. The libraries were conducted with NEBNext UltraTM RNA Library Prep Kit (NEB #E7490) on Illumina NovaSeq 6000 PE150 and purified by AMPure XP magnetic beads. Reads were processed using Trimmomatic software [17] with the default parameters to remove the adapter and low-quality reads. Next, FASTQs [18] generated from Illumina sequencing output were aligned using the STAR algorithm [19]. Read counts for individual transcripts of each sample were generated by HTSeq-count and normalized by DESeq2 algorithm to form an expression matrix and filter the differentially expressed genes. Finally, significantly differentially expressed genes were generated after the significant and FDR analysis under the following criteria: (i) |log2FC| > 1; (ii) *P* value <0.05.

### 2.8. Immunohistochemistry Analysis for NF-*κ*B Expression

Tissue sections were dewaxed and rehydrated with freshly distilled water. Endogenous peroxidase was blocked by incubating the samples with 3% H_2_O_2_ for 20 min and rinsing thrice with PBS. The paraffin block was sectioned and boiled to unmask tissue antigens. The samples were washed with PBS and blocked with 10% normal goat serum at room temperature for 30 min. Next, the slices were incubated with a primary antibody (NF-*κ*B) at 4°C overnight, followed by incubation with the peroxidase-coupled secondary antibody for 30 min at room temperature. Immunoreactivity was visualized with 3, 3'-diaminobenzidine tetrachloride (DAB). Additionally, sections were counterstained with hematoxylin and observed under the microscope (Nikon, Tokyo, Japan).

### 2.9. Western Blot Analysis

The total protein from the liver tissues was prepared using RIPA buffer (Thermo Fischer Scientific, Inc., Waltham, MA) with 1% Halt protease inhibitor cocktail and 1% Halt phosphatase inhibitor cocktails (Thermo Fischer Scientific, Inc., Waltham, MA). The concentration of extracted protein was measured using BCA Protein Assay Kit (Beyotime, Jiangsu, China). Protein samples were subjected to a 7.5%-12.5% SDS polyacrylamide electrophoresis and then electrophoretically transferred to a nitrocellulose membrane. The membrane was incubated with the primary antibodies (Supplemental material Table S1) at 4°C overnight, and then, membranes were incubated for 1 h with horseradish peroxidase-coupled secondary antibody at room temperature. Finally, the bands were scanned by the Odyssey Infrared Imaging System (LI-COR Bioscience, Lincoln, NE) and analyzed using the ImageJ2 Analysis software (US National Institutes of Health, Bethesda).

### 2.10. qPCR Assay

Total RNA in liver tissues was extracted using an RNA extraction kit (Tiangen Biotech, China) and further reverse-transcribed into cDNA using HiScript™ RT SuperMix (Vazyme Biotech, China) with Veriti Thermal Cycler (Applied Biosystems, USA). The mRNA levels were determined using the 7500 Fast Real-Time PCR System SDS software version 2.0.5 (Applied Biosystems) according to the 2^−△△Ct^ method [9]. The primers used in this study are shown in Supplemental Material Table S2. GAPDH was used as an internal control.

### 2.11. Metabolome Analysis

The liver samples and quality control (QC) were prepared as described in our previous study [9]. Chromatographic analysis was conducted using a Waters ACQUITY UPLC System (Waters Corp., Milford, MA, USA) with an Acquity UPLC®BEH C18 column (50 × 2.1 mm, 1.7 *μ*m, Waters Corp.). Mobile phase gradient elution using (a) HPLC-grade water and (b) acetonitrile (0.1% formic acid) was performed at a flow rate of 0.4 mL/min. The gradient program for solvent B was set as follows: 5-20% B from 0 to 1 min, 20-40% B from 1 to 2.5 min, 40-90% B from 2.5 to 9 min, 90-100% B from 9 to 10 min, 100% B from 10 to 14 min, and 5% B from 14 to 15 min. The autosampler was maintained at 4°C. A sample of 10 *μ*l was injected for each run. Mass spectrum (MS) was performed on a Xevo G2-S Q-TOF instrument (ACQUITY, Waters, Milford, USA). The conditions of MS were defined as previously described [9]. The metabolomic data were obtained and analyzed by Masslynx V4.1, Progenesis QI V2.4, and EZinfo V3.0 (Waters Inc.) [9]. The metabolic pathways were analyzed with MetaboAnalyst 5.0 (https://www.metaboanalyst.ca/MetaboAnalyst/).

### 2.12. Combined Transcriptome and Metabolome Analyses

Correlation analysis of transcriptome and metabolome data was performed to explore the correlation between differentially expressed genes and metabolites using RStudio with the various packages. The correlation coefficient cluster heat map, the Spearman correlation coefficient (SCC), and the cojoint KEGG pathway analysis were also analyzed using the R packages. Besides, a nine-quadrant graph was used to show the log2 (Fold change (FC)) of genes and metabolites with SCC > 0.8 and *P* value <0.05 using the R packages.

### 2.13. Statistical Analysis

Statistical analysis was performed using the software of SPSS (Ver. 17.0) for Windows. Differences between the groups were assessed using a one-way analysis of variance (ANOVA) with a Tukey's test for post hoc multiple comparisons. The data were presented as means ± standard deviation (SD). A *P* value <0.05 was considered statistically significant.

## 3. Results

### 3.1. Didymin Ameliorated LPS/D-Gal-Induced ALI in Mice

**Figure 2:**
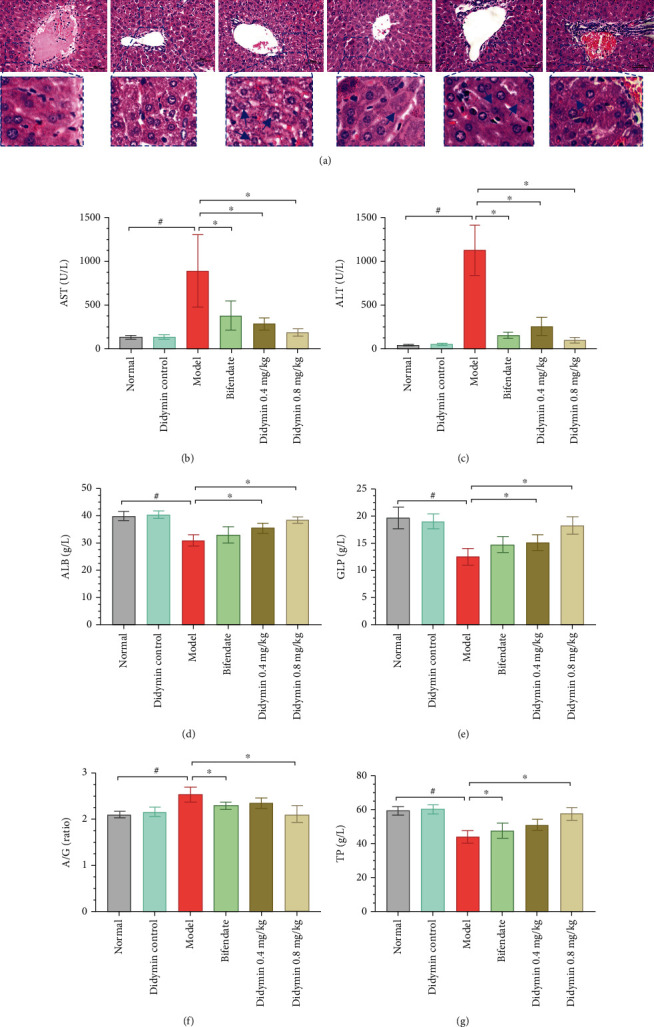
Didymin significantly alleviated LPS/D-Gal-induced ALI in mice. (a) The severity of LPS/D-Gal-induced ALI in mice was observed by H&E staining (400x); the arrow indicated necrosis. A1 to A6 represented the normal control, didymin control, model, bifendate, low, and high-dosage of didymin groups, respectively. (b–g) The levels of serum ALT, AST, ALB, GLB, A/G, and TP were detected using an automatic biochemistry analyzer. The data are presented as mean ± SD. ^#^*P* < 0.05 vs. the normal group; ^∗^*P* < 0.05 vs. the model group.

The pathological changes were assessed by H&E staining. As shown in Figure 2(a), the liver tissues of the LPS/D-Gal group revealed significant pathological alterations, including edema or hyperemia, hepatocyte necrosis, destruction of hepatic architecture, inflammatory cell infiltration, and irregularly dilated central vein. By contrast, the abnormal changes were ameliorated by didymin treatment in a dose-dependent manner, indicating that didymin strongly protected the livers from damage induced by LPS/D-Gal.

### 3.2. Didymin Protected Liver Function

Liver function was assessed by detecting the serum indexes, including AST, ALT, ALB, GLB, A/G, and TP. As shown in Figures 2(b)–2(g), AST and ALT levels were strongly elevated by LPS/D-Gal treatment in serum; on the contrary, the levels of ALB, GLB, A/G, and TP were reduced. Interestingly, these abnormalities were restored nearly to the normal levels by treatment with bifendate or didymin.

### 3.3. Didymin Alleviated Oxidative Stress and Inflammatory Response

**Figure 3:**
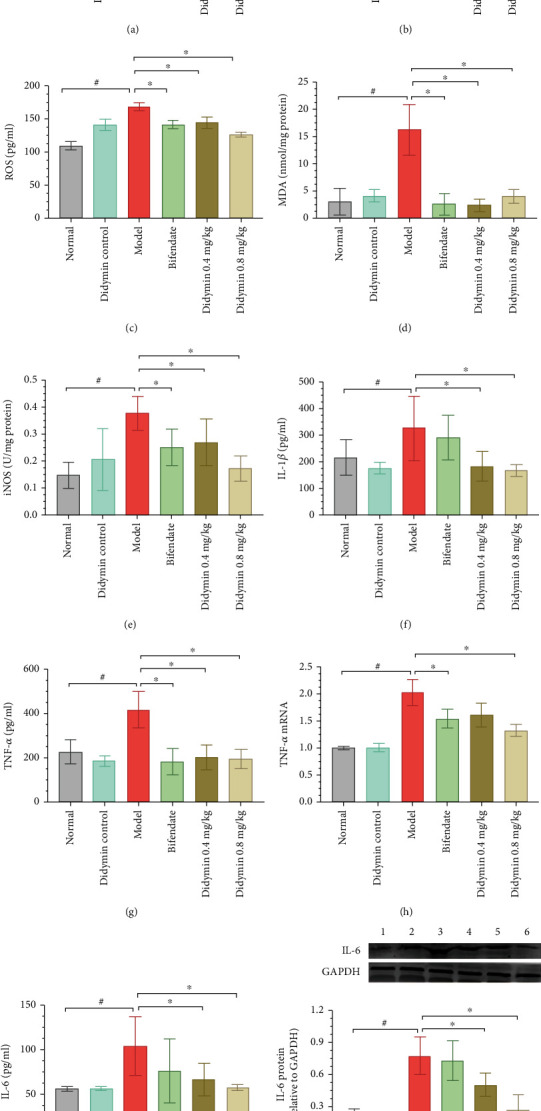
Didymin alleviated oxidative damage and inflammatory response in LPS/D-Gal-induced ALI. (a–e) The activities of GSH-Rd, SOD, ROS, MDA, and iNOS were detected by commercially available kits. (f–h) The concentrations of IL-1*β*, TNF-*α*, and IL-6 were determined by enzyme-linked immunosorbent assay. (i) The mRNA expression level of TNF-*α* was detected by RT-PCR assays. (j) The expression of IL-6 was determined using western blot. Bands 1 to 6, respectively, represent the normal control group, didymin control group, model group, bifendate-treated group, and low- and high-dosages of didymin-treated groups. The data are presented as mean ± SD. ^#^*P* < 0.05 vs. the normal group; ^∗^*P* < 0.05 vs. the model group.

As shown in Figures 3(a) and 3(b), the activities of GSH-Rd and SOD were inhibited by LPS/D-Gal treatment. Meanwhile, the levels of ROS, MDA, and iNOS were significantly increased in the model group (Figures 3(c)–3(e)). However, those abnormal changes were markedly reversed by didymin treatment. Additionally, didymin treatment significantly suppressed plasma IL-1*β*, TNF-*α* and IL-6 content, TNF-*α* mRNA level, and IL-6 protein expression (Figures 3(f)–3(j)).

### 3.4. Didymin Alleviated LPS/D-Gal-Induced Hepatocytes Apoptosis

**Figure 4:**
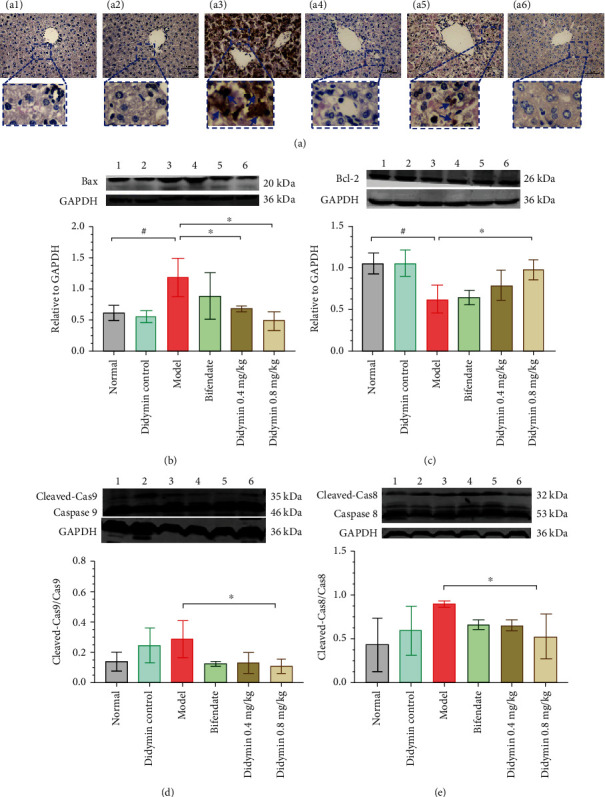
Didymin ameliorated hepatocytes apoptosis in LPS/D-Gal-induced ALI. (a) The hepatic apoptosis was detected by TUNEL staining (400x); the arrow meant hepatocyte apoptosis. (b–e) The expressions of Bcl-2 family proteins and Cleaved-caspase-9 and -8 were determined by western blot. Bands 1 to 6, respectively, represent the normal control group, didymin control group, model group, bifendate-treated group, and low- and high-dosages of didymin-treated groups. The data are presented as mean ± SD. ^#^*P* < 0.05 vs. the normal group; ^∗^*P* < 0.05 vs. the model group.

As shown in Figure 4(a), more apoptotic cells could be observed in the model group than that of the normal group; compared with the model group, the proportion of TUNEL positive regions in didymin treatment groups was significantly reduced; moreover, the expression of Bcl-2 in the didymin treatment groups was significantly increased, while the expression of Bax, Cleaved-caspase-9 (Cleaved-Cas9), and Cleaved-caspase-8 (Cleaved-Cas8) was decreased (Figures 4(b)–4(e)).

### 3.5. Transcriptome Analysis

**Figure 5:**
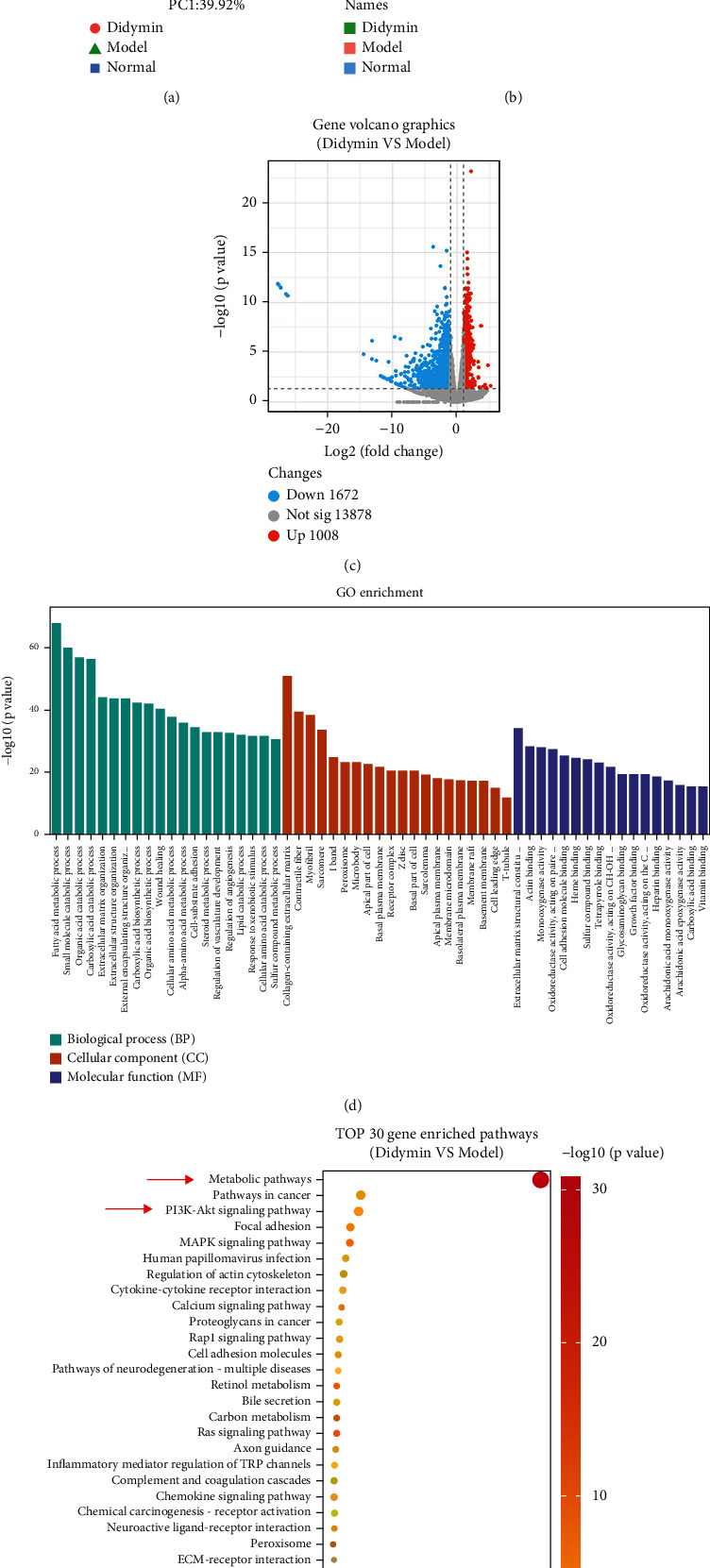
Transcriptomics analysis predicted the potential targets of didymin. (a) The PCA scores plot of normal control, model, and didymin group; the *x*-axis represents the PC1, and the *y*-axis represents PC2. (b) The heat map of DEGs. (c) The volcano plot. (d) The enriched GO analysis for the biological process (BP), cellular component (CC), and molecular function (MF). (e) The KEGG enriched pathways analysis.

The principal component analysis (PCA) revealed that the gene expression among the normal, model, and didymin-treated (didymin 0.8 mg/kg) groups was distinct. And the heat map showed that the trend of the model group was opposed to the normal control; didymin could reverse the abnormal trend to some extent, suggesting that didymin could restore the LPS/D-Gal-induced abnormal transcriptome profile (Figures 5(a) and 5(b)). RNA-seq analysis showed 2680 differently expressed genes (DEGs) (1672 down- and 1008 upregulated genes) between the model and didymin-treated groups (Figure 5(c)), which were involved in the biological process (BP), cellular component (CC), and molecular function (MF) (Figure 5(d)). Furthermore, the KEGG pathway analysis indicated more than forty signaling pathways, and the PI3K/Akt and metabolic pathways might be the most relevant ones (Figure 5(e)).

### 3.6. Didymin Inhibited the PI3K/Akt Signaling Pathway

**Figure 6:**
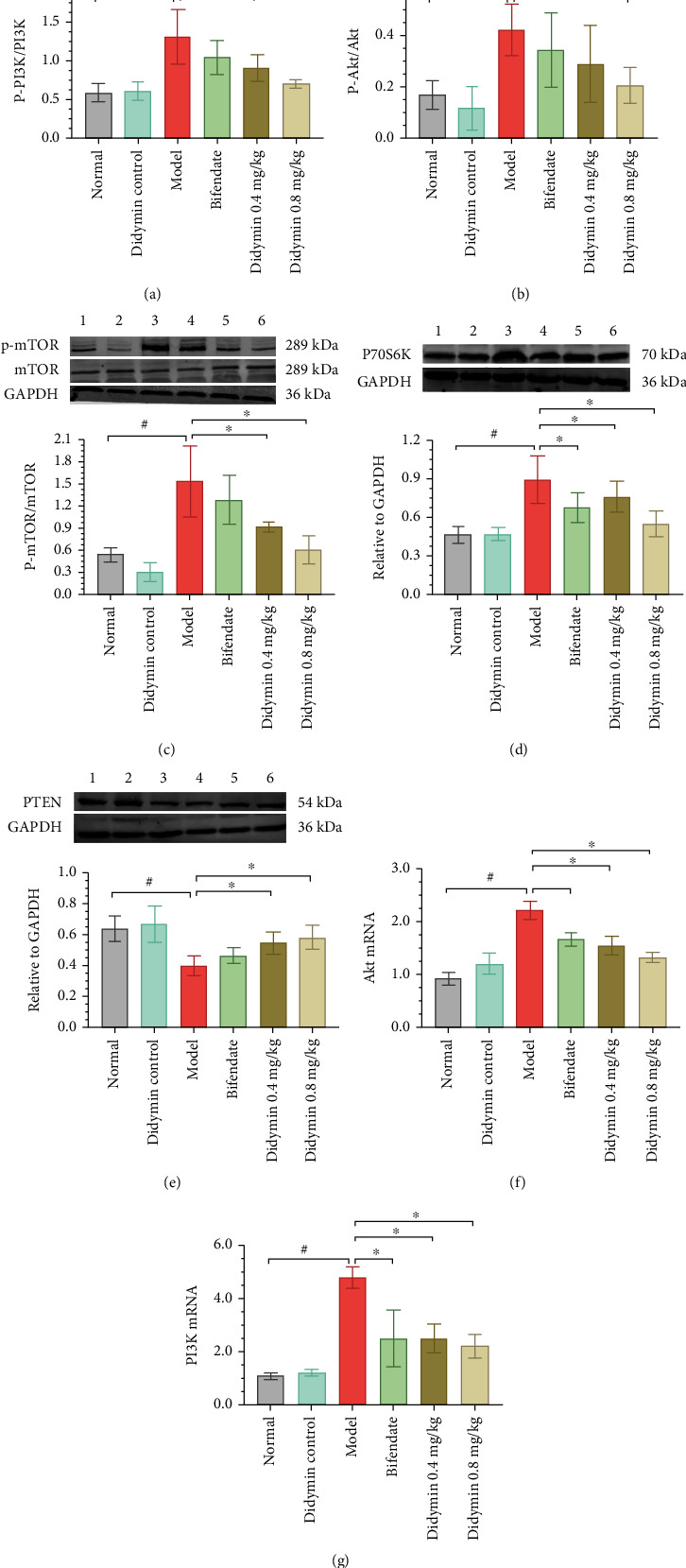
Didymin inhibited the PI3K/Akt pathway. (a–e) The expressions of p-PI3K, p-Akt, p-mTOR, P70S6K, and PTEN were determined by western blot. (f, g) The mRNA expression levels of PI3K and Akt were detected by RT-PCR assays. The bands 1 to 6, respectively, represent the normal control group, didymin control group, model group, bifendate-treated group, and low- and high-dosages of didymin-treated groups. The data are presented as mean ± SD.^#^*P* < 0.05 vs. the normal group; ^∗^*P* < 0.05 vs. the model group.

The transcriptome analysis above predicted that the PI3K/Akt signaling pathway might be the target of didymin. Therefore, the effect of didymin on this pathway was assessed. As shown in Figures 6(a)–6(e), compared with the model group, the protein expressions of P70S6K and PTEN were inhibited by didymin treatment; besides, the phosphorylations of p85, Akt, and mTOR were also significantly reduced. Moreover, the same trend was found in the mRNA expressions of Akt and PI3K; that is, they were notably decreased by didymin treatment (Figures 6(f) and 6(g)).

### 3.7. Didymin Inhibited the TLR4/NF-*κ*B Signaling Pathway

**Figure 7:**
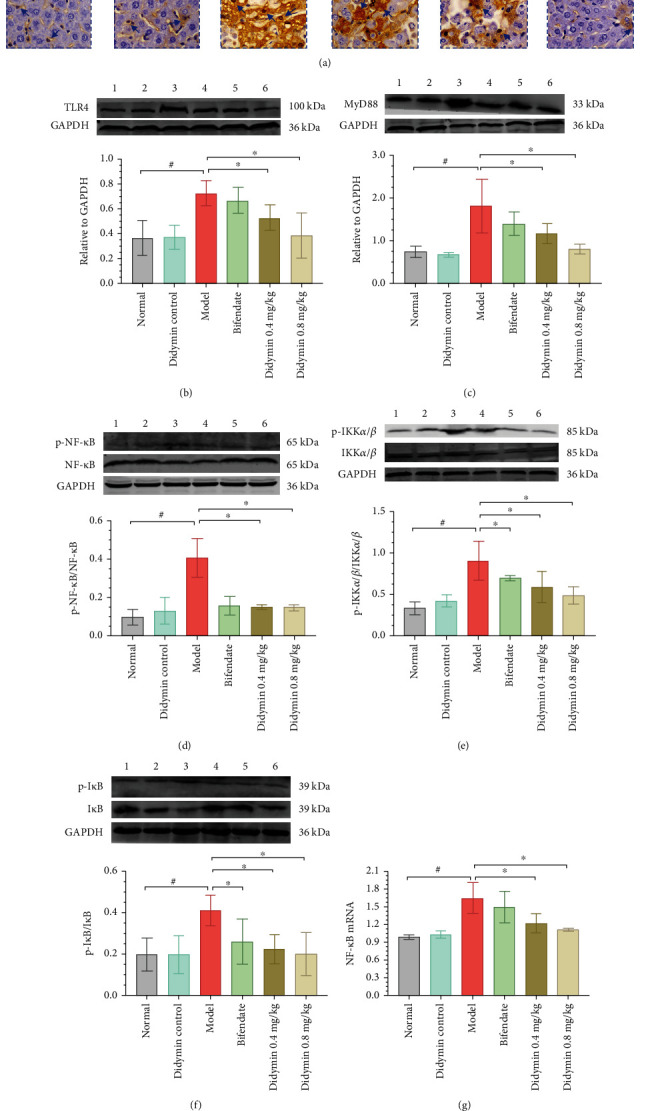
Didymin inhibited the TLR4/NF-*κ*B pathway. (a) The immunohistochemical analysis of NF-*κ*B (400x). (b–f) The expressions of TLR4, MyD88, NF-*κ*B, p-IKK*α*/*β*/IKK*α*/*β*, and p-I*κ*B*α*/I*κ*B*α* were determined by western blot. (g) The mRNA level of NF-*κ*B was detected by qPCR assays. The bands 1 to 6, respectively, represent the normal control group, didymin control group, model group, bifendate-treated group, and low- and high-dosages of didymin-treated groups. The data are presented as mean ± SD. ^#^*P* < 0.05 vs. the normal group; ^∗^*P* < 0.05 vs. the model group.

The TLR4/NF-*κ*B signaling pathway is involved in inflammation and ALI. Thus, the expressions of the TLR4/NF-*κ*B pathway-related indicators were detected in the present experiment. As shown in Figure 7(a), the immunohistochemical analysis showed that the expression level of NF-*κ*B was inhibited by bifendate or didymin. Moreover, the western blot analysis showed that didymin significantly reduced the expression levels of TLR4, MyD88, and NF-*κ*B and the phosphorylations of p-IKK*α*/*β* and p-I*κ*B*α* as well (Figures 7(b)–7(f)). Similarly, the qPCR assay also showed that the mRNA expression level of NF-*κ*B was reduced by bifendate or didymin treatment (Figure 7(g)).

### 3.8. Effect of Didymin on Metabolome in Mice with ALI

The transcriptomics analysis predicted that the metabolic pathways might be involved in the underlying mechanism of didymin against ALI. Thus, the effect of didymin on the metabolome was evaluated. The quality control (QC) analysis indicated that the Ultra-High Liquid Chromatograph-Mass Spectrum (UPLC-MS) experimental condition was high repeatability and stable, as evidenced by the similar total ion chromatogram (TIC) plot among the QC samples and a relatively tight clustering in the Principal Component Analysis (PCA) Score Plot and Loading Plot (Supplemental Figure S1).

**Figure 8:**
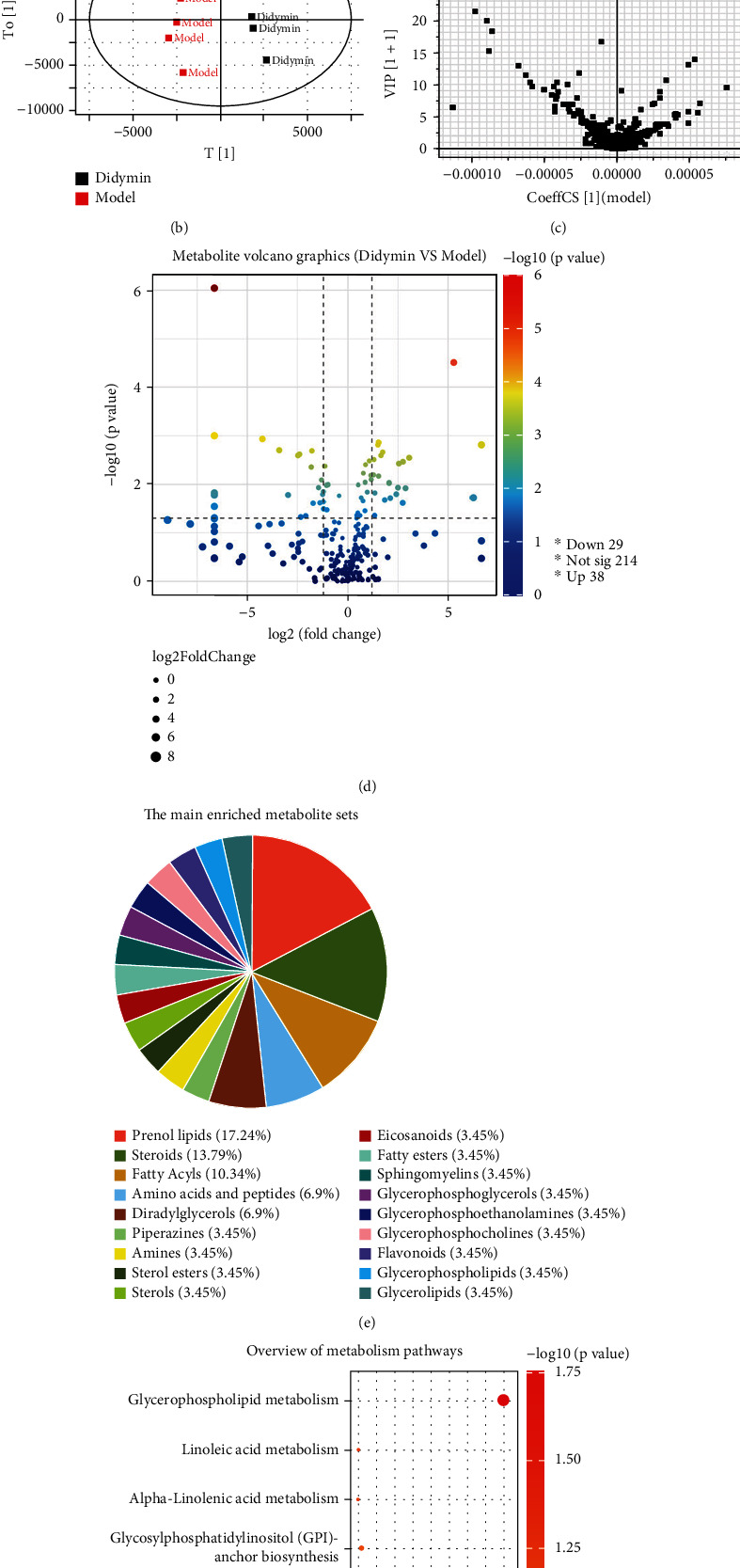
Effects of didymin on the metabolome in mice with ALI: (a) The total ion chromatogram (TIC) plot; (b) the orthogonal partial least-square discrimination analysis (OPLS-DA) plot; (c) the variable importance plot (VIP) diagram; (d) the metabolite volcano graphics; (e) the main enriched metabolite sets; (f) the enriched metabolic pathways.

As shown in Figure 8(a), LPS/D-Gal administration caused an abnormal change in the TIC plot (especially in the range of 2.5-3.5 min) compared with the normal group; however, didymin treatment could reverse the abnormal change, suggesting that didymin could ameliorate LPS/D-Gal-induced abnormal transcriptome profile to some extent. The Orthogonal Partial Least-Square Discrimination Analysis (OPLS-DA) is a weighted average of the original scores, providing a good summary and displaying the separation between groups. As shown in Figure 8(b), the OPLS-DA revealed an obvious separation between the model and didymin groups, which was confirmed by the variable importance plot (VIP) (Figure 8(c)). Further analysis indicated 67 differently expressed metabolites (DEMs) between the model and didymin groups (Figure 8(d)), which were mainly enriched in several metabolite sets, such as steroids, prenol lipids, piperazines, and fatty acyls (Figure 8(e)) and clustered into seven metabolism-related pathways, including glycerophospholipid metabolism, linoleic acid metabolism, alpha-linolenic acid metabolism, glycosylphosphatidylinositol- (GPI-) anchor biosynthesis, sphingolipid metabolism, arachidonic acid metabolism, and steroid biosynthesis. Interestingly, the glycerophospholipid metabolism pathway might be the most relevant one among the metabolic pathways (*P* < 0.05 and impact >0.1) (Figure 8(f)).

### 3.9. Integrative Analysis of the Transcriptome and Metabolome

**Figure 9:**
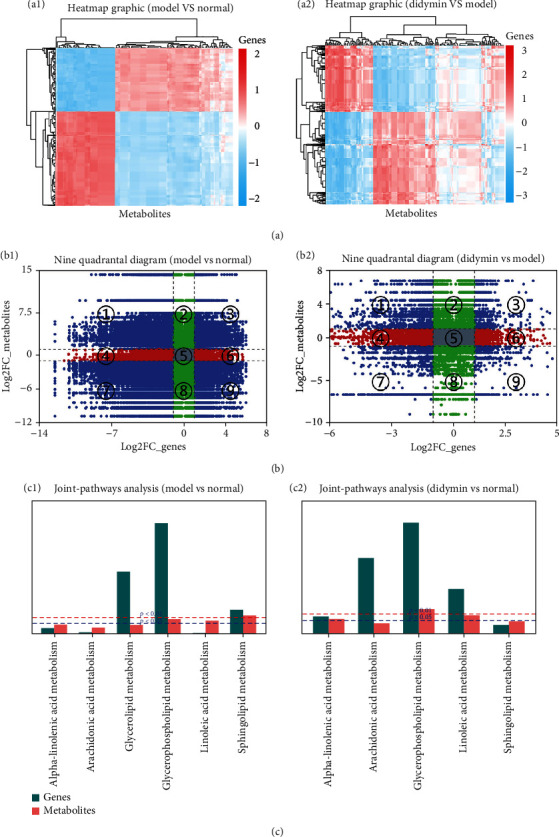
Correlation analysis of the transcriptome and metabolome. (a) The correlation heat map. (b) Nine quadrant diagram showing the correlation of genes and metabolites; the *x*-axis represents the log2 ratio of gene, and the *y*-axis represents the log2 ratio of metabolites; black-dotted lines represent the different thresholds. (c) KEGG enrichment analysis of the differentially expressed genes (dark cyan column) and differentially expressed metabolites (red column); the blue line represents the selected gene and metabolic pathway at *p* value <0.05, and the red line represents the selected gene and metabolic pathway at *P* value <0.01.

To further elucidate the correlation between transcriptome and metabolome, the correlation coefficient was calculated using R packages with the Spearman method. As shown in Figure 9(a), the heat map graphics revealed that many genes and metabolites were negatively or positively correlated, suggesting that the metabolism was likely affected by the transcriptome. The nine-quadrant diagrams [20] were consistent with the result of the heat map and provided more detailed information (Figure 9(b)). The correlation coefficient data (|*r*| > 0.80) were divided into 1-9 quadrants: the 1 and 9 quadrants indicated the negative correlation between the genes and metabolites; while the quadrants 3 and 7 suggested that they were positively correlated; the 5 quadrant meant no significance, and the rest of quadrants suggested a partial correlation. These results from the heat map, and the nine-quadrant diagrams indicated that many genes might play direct or indirect regulatory roles in the alterations in the corresponding metabolites. In addition, the cojoint KEGG pathway enrichment analysis of transcriptome and metabolome was performed as previously described [21]. As shown in Figure 9(c), the genes and the corresponding metabolites were enriched in 5 identical metabolic pathways between the didymin and model groups. And the glycerophospholipid metabolism was the common pathway for genome enrichment and metabolome enrichment (*P* < 0.05).

**Figure 10:**
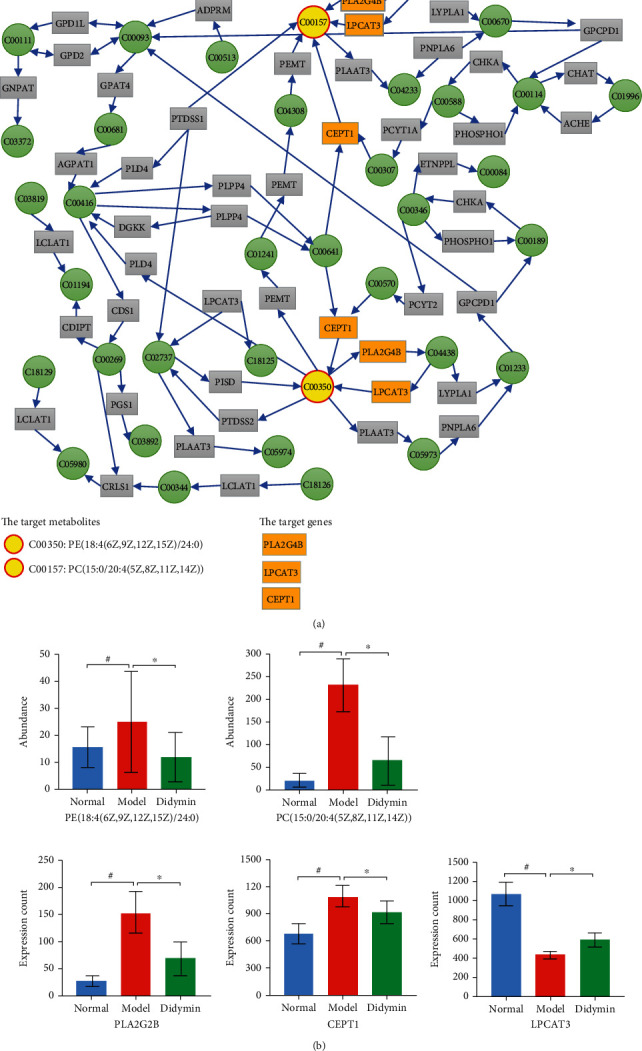
The integrative analysis of transcriptome and metabolome was conducted by MetaboAnalyst 5.0. (a) The glycerophospholipid metabolism pathway. (b) The contents of PE (18 : 4 (6Z,9Z,12Z,15Z)/24 : 0) and PC (15 : 0/20 : 4 (5Z,8Z,11Z,14Z)) and the expression levels of PLA2G4B, LPCAT3, and CEPT1. The data are presented as mean ± SD. ^#^*P* < 0.05 vs. the normal group; ^∗^*P* < 0.05 vs. the model group.

To understand more detailed information on how didymin regulated the glycerophospholipid metabolism, the integrative analysis of transcriptome and metabolome was further conducted by the MetaboAnalyst 5.0 database (https://www.metaboanalyst.ca/MetaboAnalyst/). As shown in Figure 10, the analysis indicated that didymin regulated PLA2G4B, LPCAT3, and CEPT1 expression and, as a result, affected the synthesis of PE (18 : 4 (6Z,9Z,12Z,15Z)/24 : 0) and PC (15 : 0/20 : 4 (5Z,8Z,11Z,14Z)), ultimately regulating the glycerophospholipid metabolism pathway. Additionally, the quantitive analysis confirmed that didymin decreased the expression levels of PLA2G4B and CEPT1, while it increased LPCAT3 level to some extent; meanwhile, didymin significantly decreased the contents of PE (18 : 4 (6Z,9Z,12Z,15Z)/24 : 0) and PC (15 : 0/20 : 4 (5Z,8Z,11Z,14Z)). These data further verified the Joint-Pathway Analysis.

## 4. Discussion

In this study, the ALI model in mice was induced by LPS/D-Gal, which has been widely used to assess the hepatoprotective effects of drugs [22]. Compared to the LPS/D-Gal model group, didymin treatment significantly ameliorated the pathologic damage, as evidenced by the alleviated hyperemia, necrosis, and inflammatory cell infiltration in the H&E staining. Interestingly, didymin exerted a strong protective effect on ALI in mice, which was similar to that of bifendate. In view of the lower dose of didymin, the protective effect of didymin may be better than that of bifendate. Besides, liver function-related biomarkers were detected. The data showed that didymin and bifendate could significantly reduce the aminotransferase activity compared to the model control, suggesting that didymin could improve hepatic function. Since hepatocyte apoptosis is also one of the mechanisms involved in liver damage, we also focused on the effects of didymin on hepatocyte apoptosis. The TUNEL staining revealed numerous positive apoptotic cells in the LPS/D-Gal model group, and didymin administration significantly reversed these abnormal changes. Furthermore, compared to the LPS/D-Gal model control, didymin treatment remarkably decreased the ratios of Bax/Bcl-2, Cleaved-caspase-8/caspase-8, and Cleaved-caspase-9/caspase-9, which suggested that the possible mechanism by which didymin attenuated hepatocyte apoptosis might be related to the regulation of the expression of Bcl-2 and caspase family proteins. Briefly, the results of the pathologic examination and hepatocyte apoptosis detection demonstrated that didymin could ameliorate LPS/D-Gal-induced ALI in mice.

Oxidative stress has been regarded as a crux influence cause of hepatic injury. The imbalance of the oxidative and antioxidant systems easily leads to oxidative damage to the hepatocytes, playing an important role in liver injury. In this study, the activities of SOD and GSH-Rd were drastically decreased in the LPS/D-Gal group, while the level of MDA was strongly elevated; however, these abnormal changes were largely reversed by didymin treatment, suggesting that didymin could recruit the antioxidative defense system and, as a result, decrease oxidative damage. In addition, excessive inflammation is also one of the risk factors that induces and accelerates liver injury. The current study showed that didymin significantly reduced the levels of iNOS, TNF-*α*, IL-1*β*, and IL-6, suggesting that didymin had the capacity of anti-inflammation. Next, the TLR4/NF-*κ*B signaling pathway was detected to investigate the potential mechanism of didymin against inflammation. NF-*κ*B belongs to the Rel protein family, and the heterodimer composed of p65/p50 is the main biologically active form, which combines with I*κ*B*α* to form a trimer (inactive form). IKK*α* can phosphorylate I*κ*B*α* and I*κ*B*β*, which results in their ubiquitination and the activation of TLR4/NF-*κ*B signaling [23], ultimately leading to numerous inflammatory cytokines over-secretion. A previous study indicated that didymin could inhibit the activation of NF-*κ*B and exert anti-inflammatory actions, preventing hyperglycemia-induced human umbilical endothelial cell dysfunction and death [24]. In this study, didymin treatment markedly suppressed the expressions of TLR4, MyD88, NF-*κ*B, and the phosphorylation of IKK*α*/*β* and I*κ*B*α*, which was consistent with the previous research [24]. Our data indicated that didymin could inhibit the TLR4/NF-*κ*B pathway and, consequently, reduce inflammation.

Next, the underlying mechanisms of didymin against liver injury were further investigated by transcriptome analysis. Transcriptome is the collection of all transcripts within a cell, including mRNA, rRNA, tRNA, and other noncoding RNAs. Unlike conventional PCR experiments, transcriptome sequencing can comprehensively obtain almost all transcripts of a specific organ or tissue in a certain state and reflect their expression levels, predicting the pathogenesis of a disease or the therapeutic mechanism of drugs as a whole. In the current study, the RNA-seq revealed more than two thousand differently expressed genes (DEGs) between the model and didymin-treated groups, and these DEGs were enriched into many pathways. Interestingly, the metabolic and PI3K/Akt pathways likely were the most relevant targets, as indicated by the KEGG pathways analysis.

Since the transcriptomics analysis suggested the key role of the PI3K/Akt pathway in didymin treating liver injury, the effect of didymin on this signaling pathway was then assessed. Numerous studies have demonstrated that the PI3K/Akt pathway is involved in the onset and development of liver injury [25]. Akt receives the biological signals transmitted and phosphorylated and activated by PIP3, causing the activation or inhibition of downstream signaling pathways to regulate cell proliferation, differentiation, migration, and apoptosis [26]. Like many previous studies [27, 28], the current study found that LPS/D-Gal administration resulted in the PI3K/Akt pathway activation and induced cell apoptosis. On the contrary, didymin treatment significantly decreased the phosphorylations of PI3K, Akt and mTOR, and the expression of P70S6K. These results suggested that didymin significantly inhibited the PI3K/Akt signaling pathway, which was consistent with the prediction of the transcriptome analysis.

Besides, the transcriptome analysis also predicted that the metabolic pathways might be involved in the protective effects of didymin on ALI. Thus, an untargeted metabolomics approach was employed to define the changed metabolic pathways and find clues to uncover the potential mechanisms. The mass spectrum showed that LPS/D-Gal administration led to a significant change in the global liver metabolic profiles. Interestingly, didymin treatment could restore the abnormal change nearly to the normal level. Further analysis revealed 67 differently expressed metabolites (DEMs) between the model and didymin groups, which were enriched into seven metabolism-related pathways. Among the significantly changed pathways, the glycerophospholipid metabolism pathway might be the most important (*P* < 0.05 and impact >0.1).

To understand how didymin regulated metabolism, we conducted an in-depth analysis of the transcriptome and metabolome. We found that the DEGs and DEMs were closely related; many genes might play direct or indirect regulatory roles in the alterations in the corresponding metabolites. And the evidence also indicated that didymin regulated the metabolism of many substances *in vivo* by influencing the expression of genes. In addition, the Joint-Pathway Analysis suggested that didymin could decrease the synthesis of PE (18 : 4 (6Z,9Z,12Z,15Z)/24 : 0) and PC (15 : 0/20 : 4 (5Z,8Z,11Z,14Z)) by regulating PLA2G4B, LPCAT3, and CEPT1 expression, ultimately inhibiting the glycerophospholipid metabolism pathway. It has been confirmed that phosphoethanolamines (PEs) and phosphocholines (PCs) are the key phospholipids of cell membranes [17], which are involved in the synthesis of the characteristic bilayer structure of cells and regulate membrane integrity sensitivity to oxidative stress. In the current study, didymin significantly inhibited the glycerophospholipid metabolism pathway, decreasing the content of the PE and PC metabolites in the liver. These results suggested that the protective effects of didymin on hepatocytes were also associated with the glycerophospholipid metabolism pathway.

**Figure 11:**
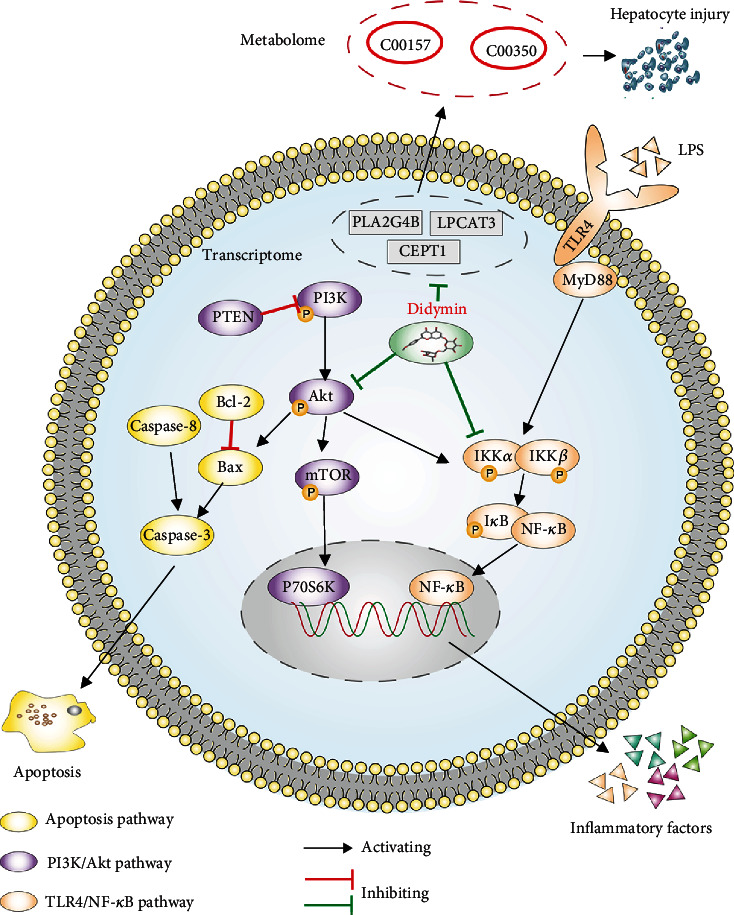
Didymin alleviates LPS/D-Gal-induced ALI in mice by inhibiting the PI3K/Akt and TLR4/NF-*κ*B signaling pathways as well as the glycerophospholipid metabolism pathway.

In conclusion, didymin can meliorate LPS/D-Gal-induced ALI in C57BL/6J mice by regulating the PI3K/Akt and TLR4/NF-*κ*B signaling pathways and the glycerophospholipid metabolism pathway (Figure 11). Our study probably provides a new perspective on didymin as a potential agent for the treatment of ALI.

## Data Availability

The authors declare that they have no known competing financial interests or personal relationships that could have appeared to influence the work reported in this paper.

## Additional Points

The supplemental data were provided as the supplementary material; the transcriptome data were published at https://www.ncbi.nlm.nih.gov/geo/query/acc.cgi?acc=GSE190823; the metabolome data could be found at http://www.ebi.ac.uk/metabolights/MTBLS3929. And the other raw data, such as the raw bands of the western blot, were submitted as a pdf of raw data of didymin.

## Supplementary Materials

Supplementary Materials Supplemental material provided the primary antibody for western blot analysis (Table S1), the sequences of primers for qPCR (Table S2), the investigation for the QC stability (Figure. S1), and the differential metabolites between groups (Table S3 and S4).
