# Supplementary material for: Integrative Analysis of Transcriptome and Metabolome to Illuminate the Protective Effects of Didymin against Acute Hepatic Injury
Source: Mediators Inflamm. 2023 Jan 12;2023:6051946. doi: 10.1155/2023/6051946 (PMC9851790; doi:10.1155/2023/6051946)
Supplement: Supplementary Materials — Supplemental material provided the primary antibody for western blot analysis (Table S1), the sequences of primers for qPCR (Table S2), the investigation for the QC stability (Figure. S1), and the differential metabolites between groups (Table S3 and S4). [file 6051946.f1.pdf]

Supplemental Material

Table S1 The primary antibody for Western blot analysis

Antibody	Information
PI3K P110	1:1000, Cell Signaling Technology Inc
PI3K P85	1:1000, Cell Signaling Technology Inc
Akt	1:1000, Cell Signaling Technology Inc
mTOR	1:800, Cell Signaling Technology Inc
P70S6K	1:1000, Cell Signaling Technology Inc
PTEN	1:1000, Cell Signaling Technology Inc
p-PI3K	1:500, Cell Signaling Technology Inc
p-Akt	1:500, Cell Signaling Technology Inc
p-mTOR	1:500, proteintech
TLR4	1:500, Cell Signaling Technology Inc
MyD88	1:1000, Cell Signaling Technology Inc
NF-κB	1:1000, Cell Signaling Technology Inc
IKKα/β	1:1000, Cell Signaling Technology Inc
p-IκB	1:500, Cell Signaling Technology Inc
Bax	1:1000, Cell Signaling Technology Inc
Bcl-2	1:800, abcam
Caspase-8	1:1000, proteintech
Caspase-9	1:1000, proteintech
IL-6	1:500, proteintech
GAPDH	1:1000, proteintech

Table S2 The sequences of primers

Primer	Sequence（5'-3')	Anti-sequence（5'-3')
GAPDH	CCTCGTCCCGTAGACAAAATG	TGAGGTCAATGAAGGGGTCGT
NF-κB	AAGCACAGATACCACCAAGACAC	CGCACTGCATTCAAGTCATAGTC
TNFα	CCCTCACACTCACAAACCACC	CTTTGAGATCCATGCCGTTG
Akt	TTTGGGAAGGTGATTCTGGTG	CAGGACACGGTTCTCAGTAAGC
PI3K	CAAACCACCCAAGCCCACTA	AGGTCCCATCAGCAGTGTCTC

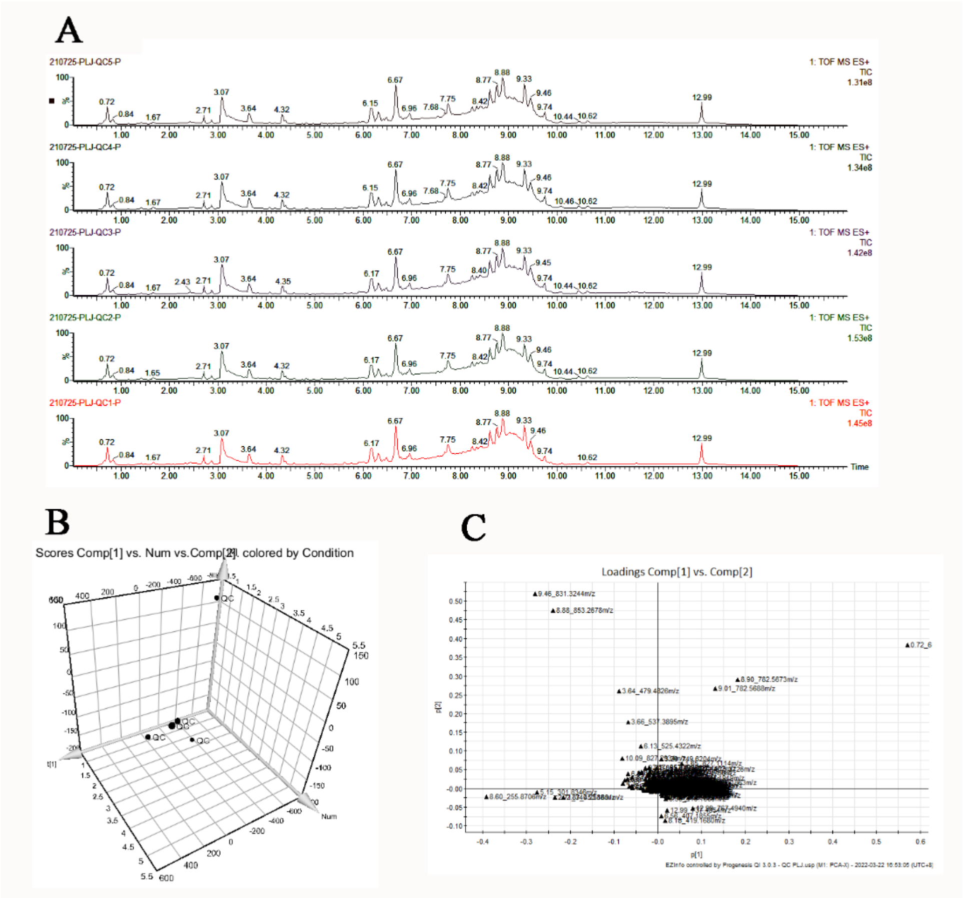

Figure. S1 The investigation for the QC stability

A: Total ion chromatogram (TIC) of QC; B: Principal Component Analysis (PCA) Score Plot; (C) Loading Plot

Table S3 The differential metabolites between the normal and model groups.

HMDB ID	Match	KEGG	m/z	RT (min)	A nova (p)	Trend[a]
HMDB0034081	Brassinolide	C11049	481.3513	0.72	1.3E-10	up
HMDB0006737	CE(22:2(13Z,16Z))	C02530	705.6532	3.09	1.5E-04	up
HMDB0012105	SM(d18:1/23:0)	C00550	801.6825	0.60	4.8E-04	down
HMDB0005042	Aripiprazole	C12564	448.1559	4.07	9.9E-04	down
HMDB0007949	PC(15:0/20:4(5Z,8Z,11Z,14Z))	C00157	768.5526	2.46	1.0E-03	down
HMDB0014681	Vincristine	C07204	825.4094	8.62	8.0E-03	down
HMDB0014531	Procyclidine	C07378	288.2332	6.31	9.7E-03	down
HMDB0009211	PE(18:4(6Z,9Z,12Z,15Z)/24:0)	C00350	824.6183	1.65	8.2E-01	up

a: the trend in model group

Table S4 The differential metabolites between the model group and didymin-treated group.

HMDB ID	Match	KEGG	m/z	RT (min)	Anova (p)	Trend[a]
HMDB0007949	PC(15:0/20:4(5Z,8Z,11Z,14Z))	C00157	768.553	2.46	0.001	down
HMDB0034081	Brassinolide	C11049	481.351	0.72	0.002	up
HMDB0012105	SM(d18:1/23:0)	C00550	801.683	0.60	0.012	down
HMDB0005042	Aripiprazole	C12564	448.156	4.07	0.017	down
HMDB0006737	CE(22:2(13Z,16Z))	C02530	705.653	3.09	0.019	up
HMDB0014681	Vincristine	C07204	825.409	8.62	0.022	up
HMDB0014531	Procyclidine	C07378	288.233	6.31	0.039	up
HMDB0009184	PE(18:4(6Z,9Z,12Z,15Z)/14:0)	C00350	684.462	6.13	0.762	down

a: the trend in didymin-treated group

Raw data of this study

Fig. 2 Didymin significantly alleviated LPS/D-Gal-induced ALI in mice. (A) The severity of LPS/D-Gal-induced ALI in mice was observed by H&E staining;

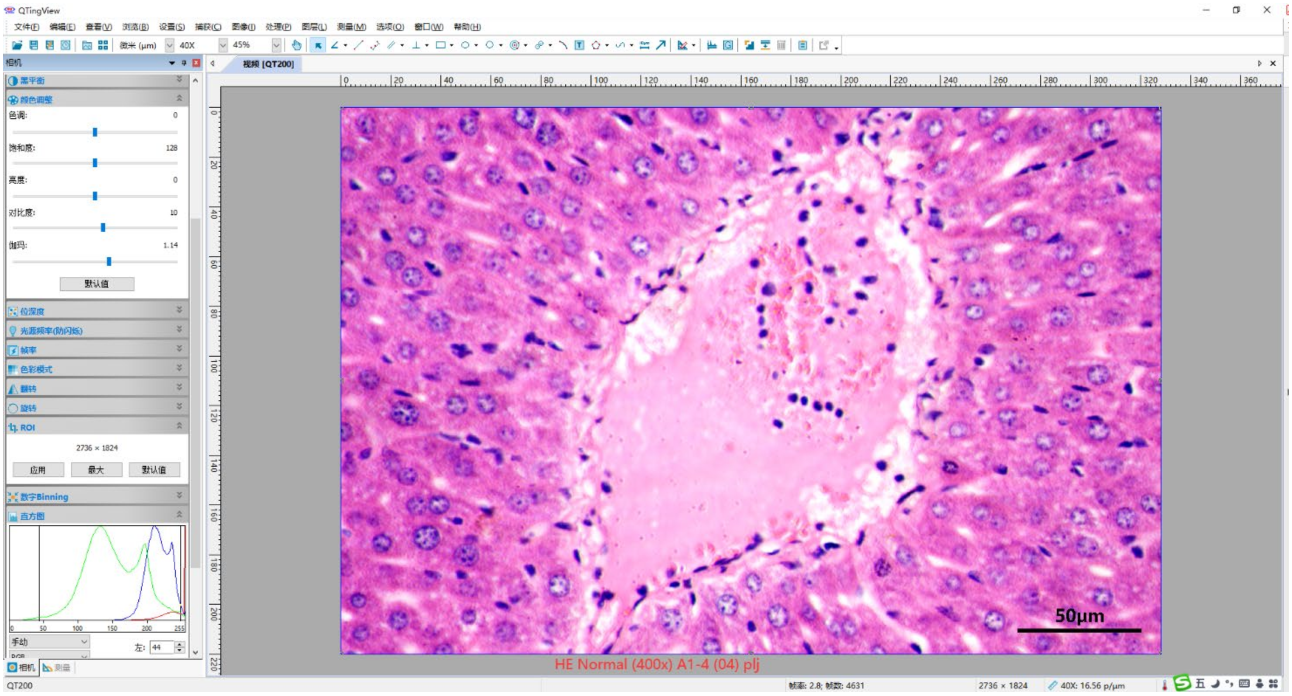

Normal group (400×)

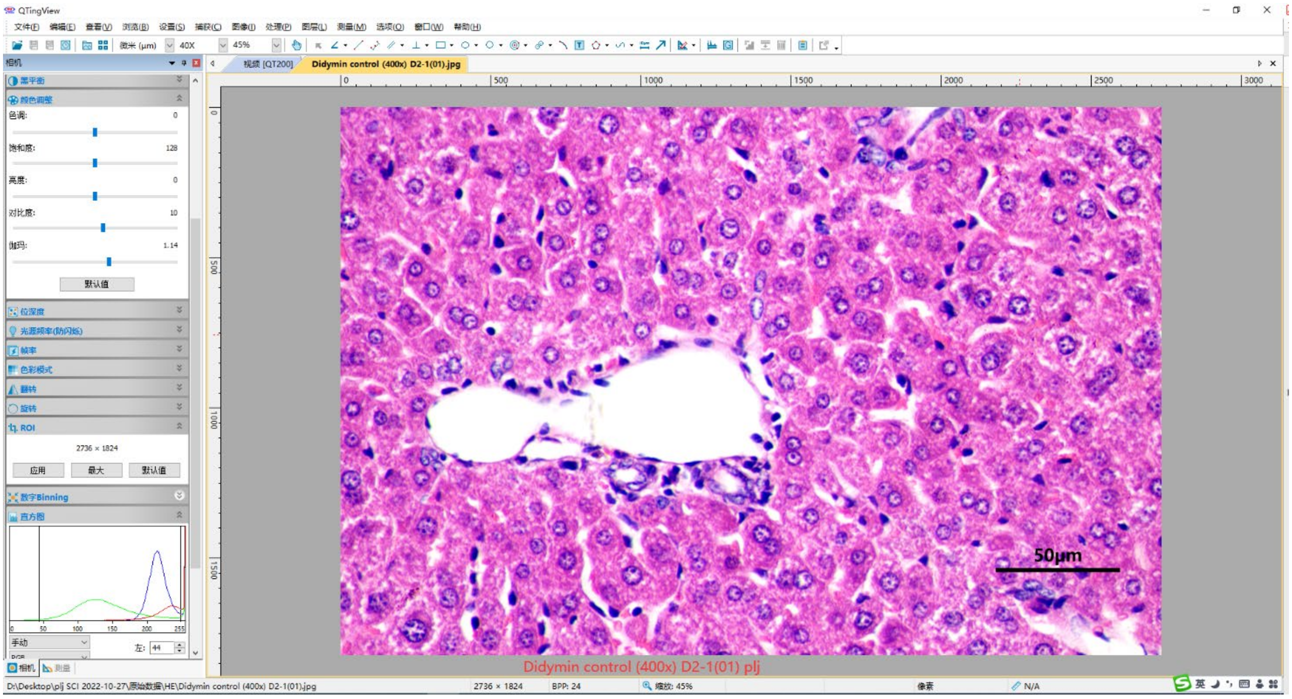

Didymin control group (400×)

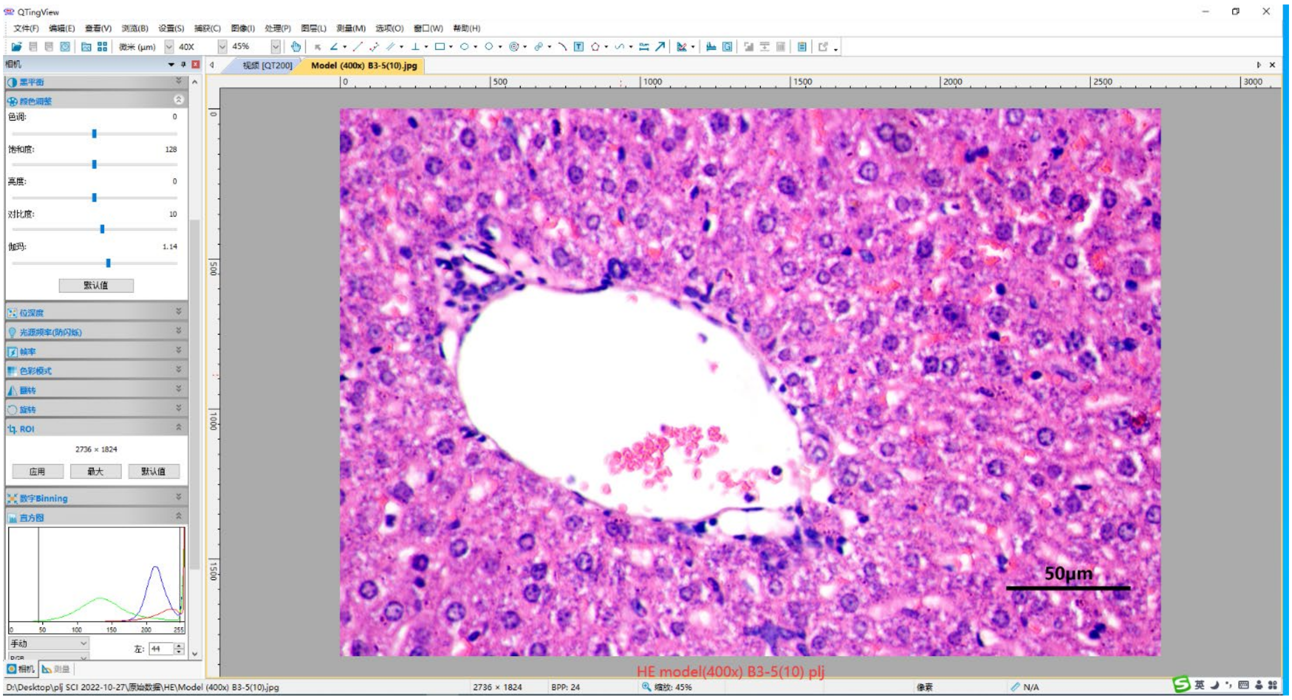

Model group (400×)

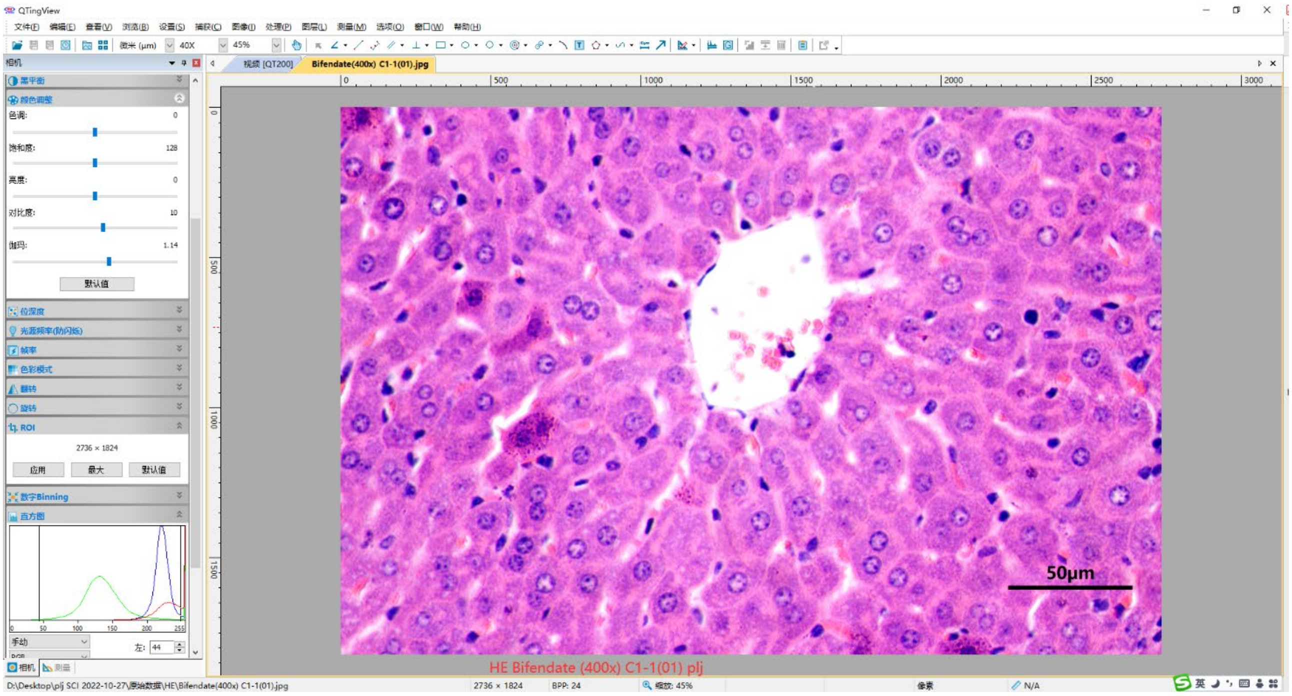

Bifendate group (400×)

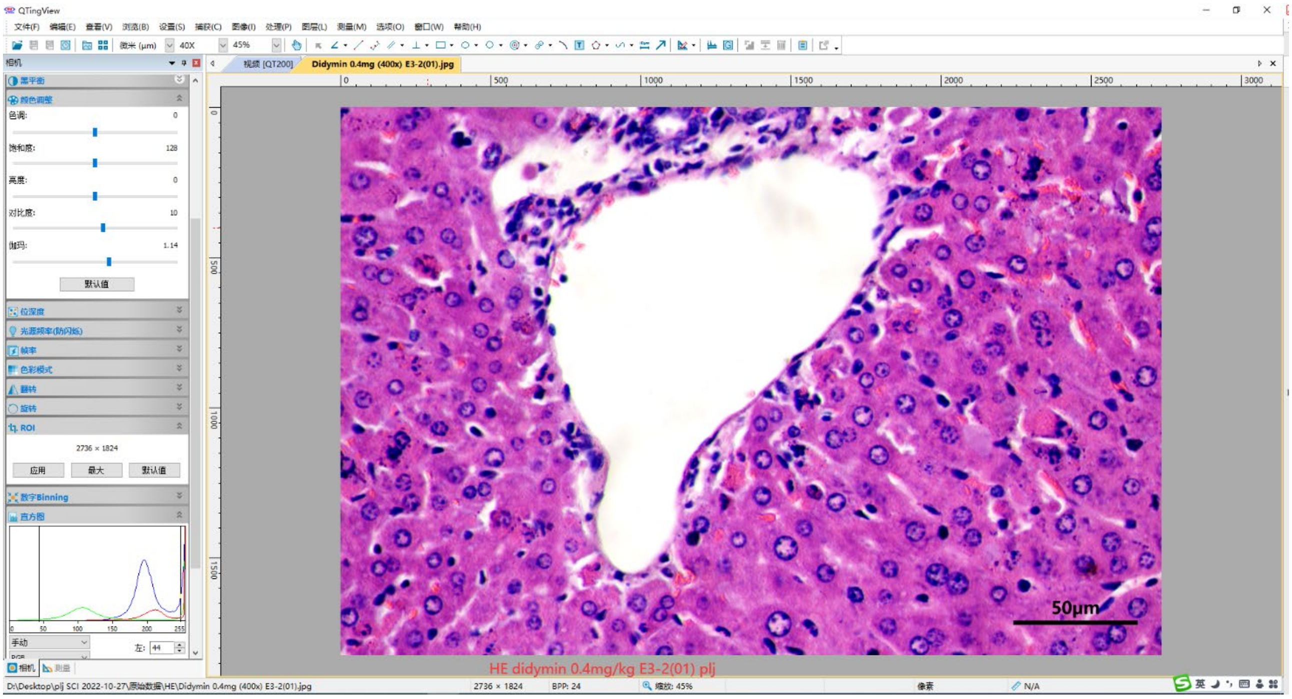

Didymin treated-group (0.4 mg/kg) (400×)

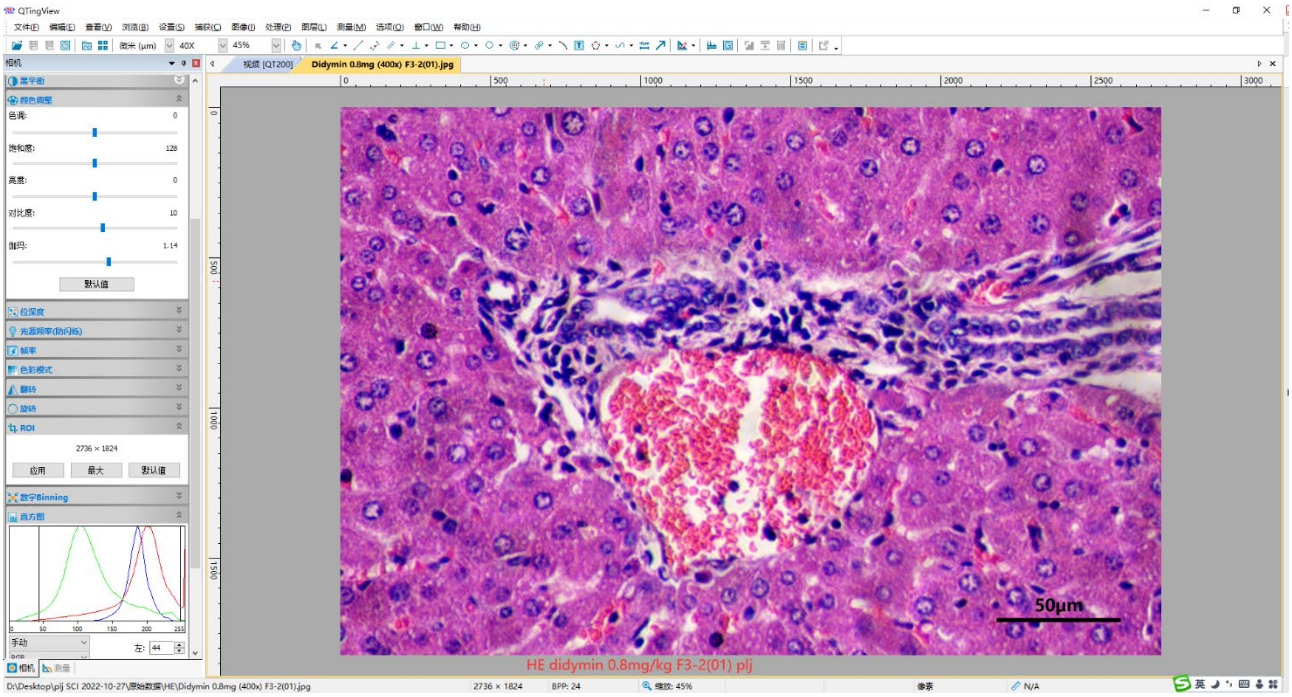

Didymin treated-group (0.8 mg/kg) (400×)

Fig. 3 IL-6 protein expression

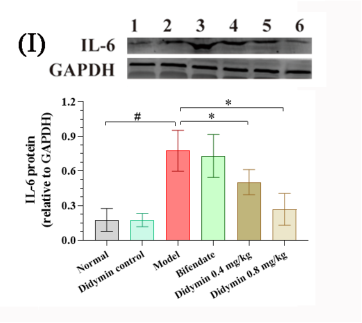

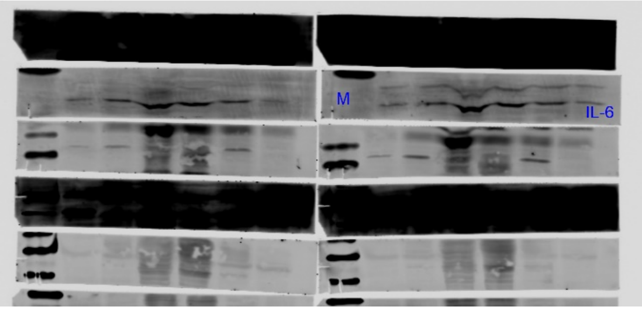

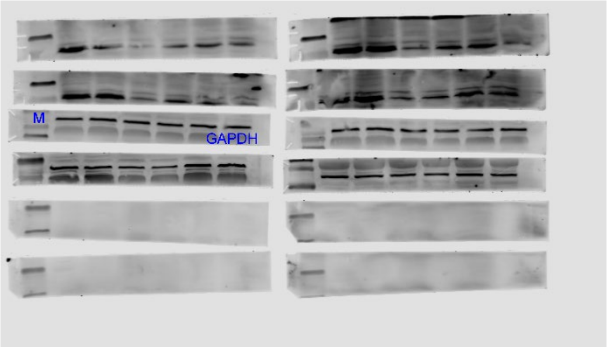

Fig. 4 Didymin ameliorated hepatocytes apoptosis in LPS/D-Gal-induced ALI. (A) The hepatic apoptosis was detected by TUNEL staining

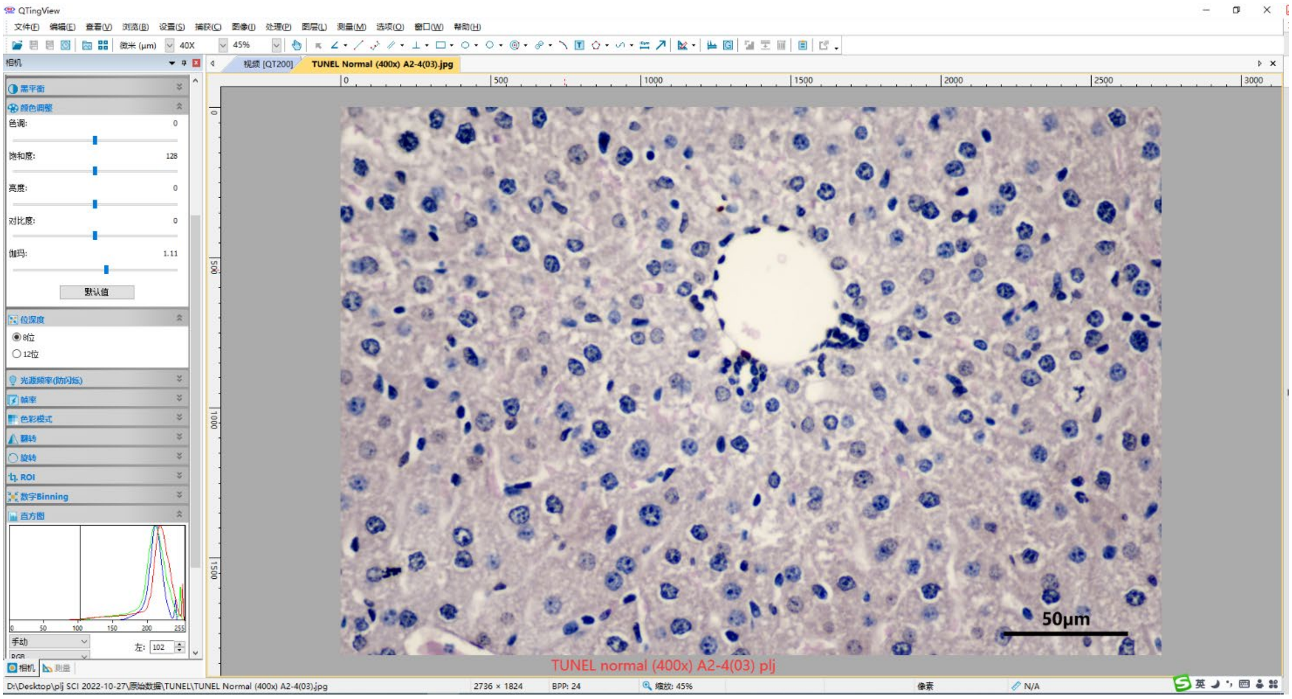

Normal group (400×)

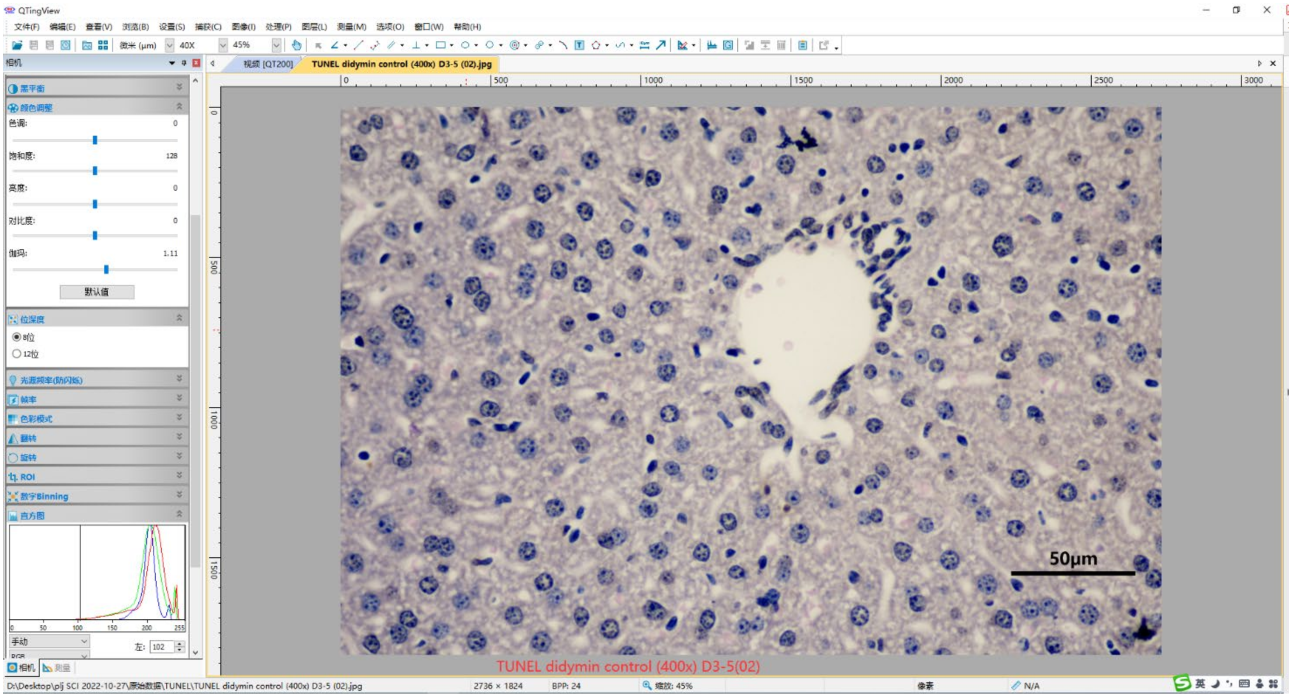

Didymin control group (400×)

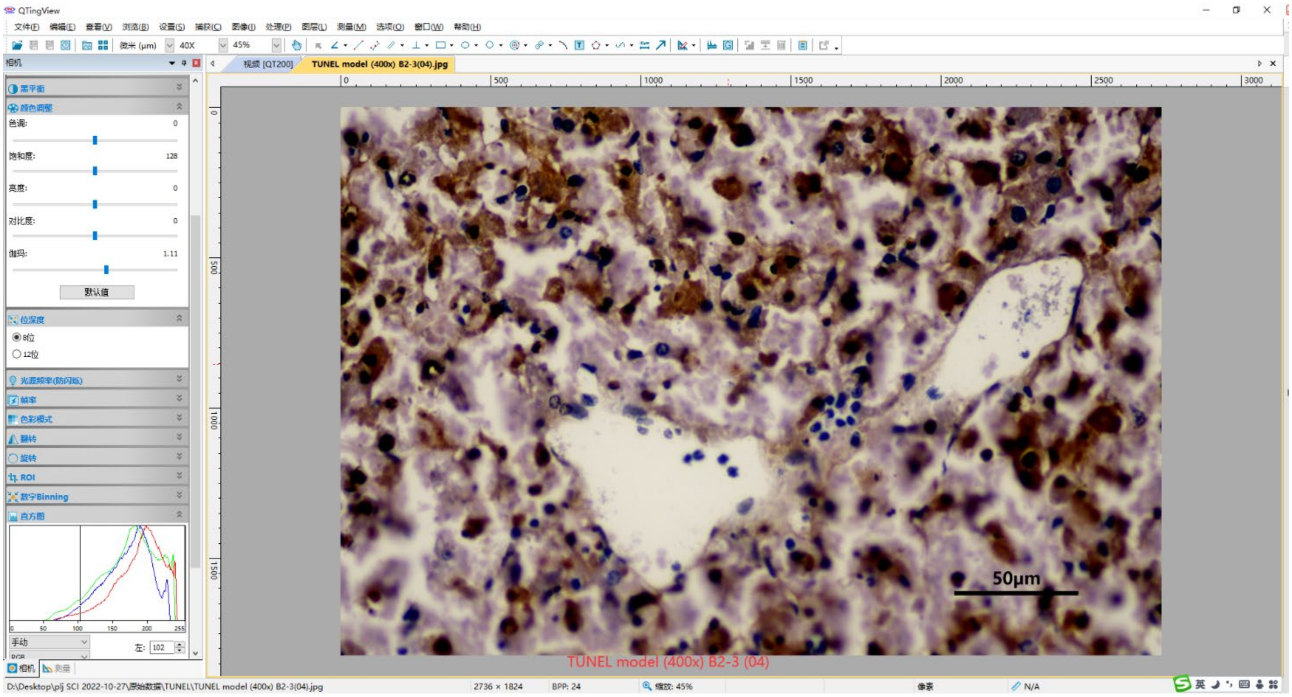

Model group (400×)

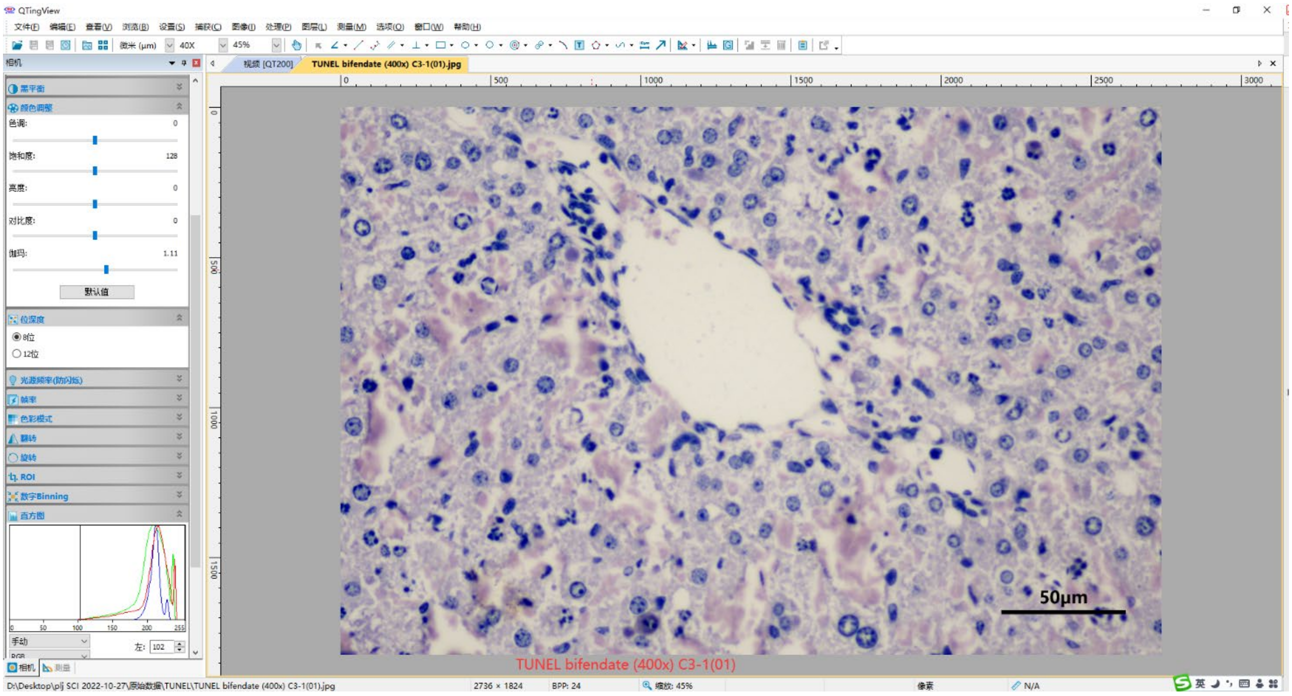

Bifendate group (400×)

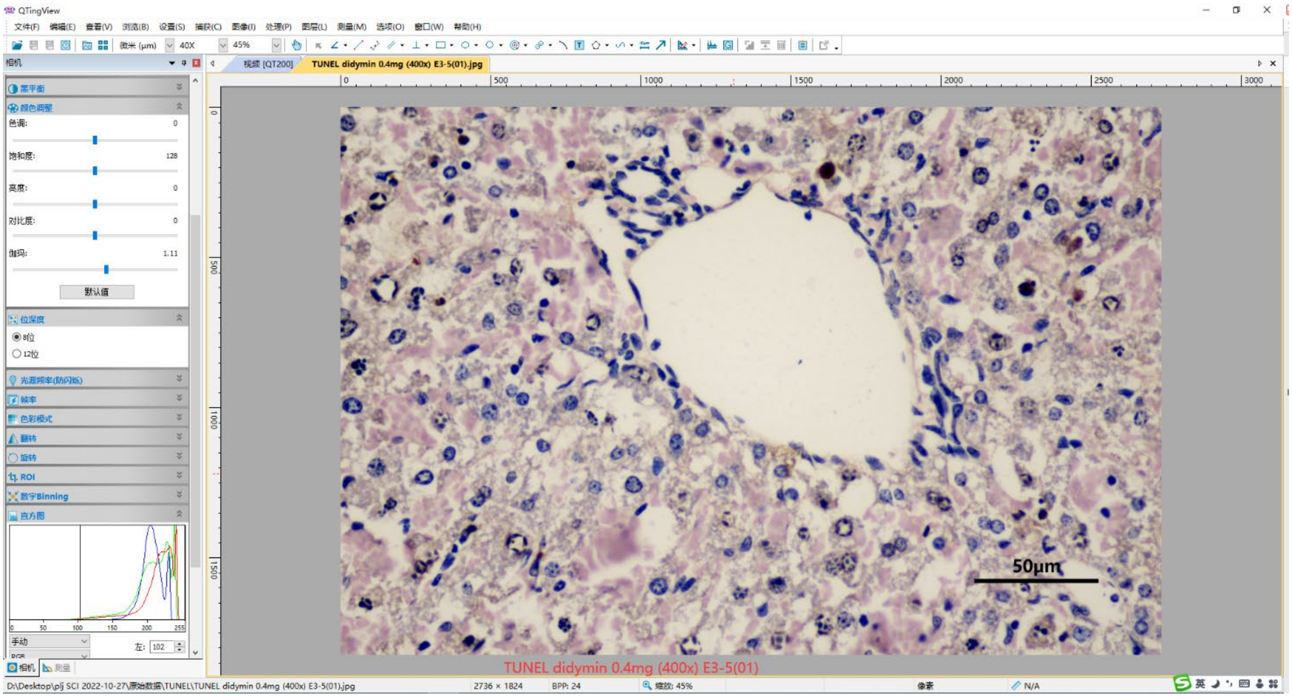

Didymin-treated group (0.4mg/kg) (400×)

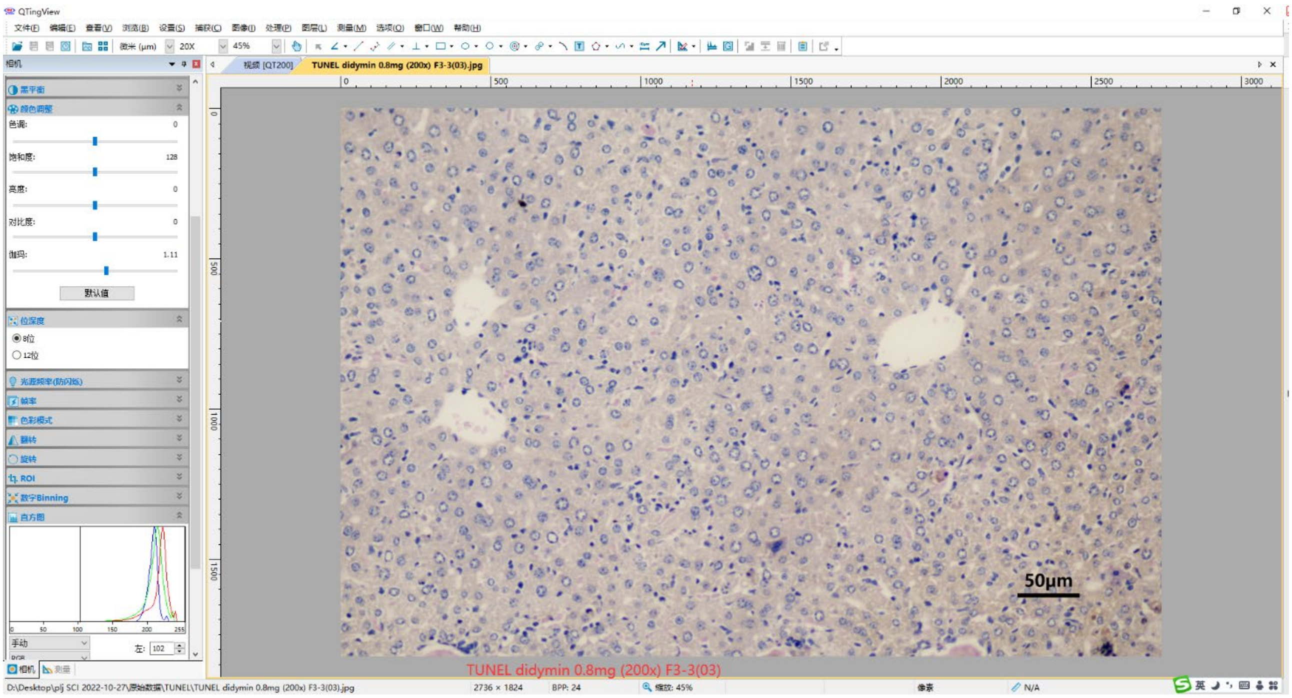

Didymin-treated group (0.8mg/kg) (200x)

Figure 4 The protein expression of Bax, Bcl-2, Caspase-8 and Caspase-9

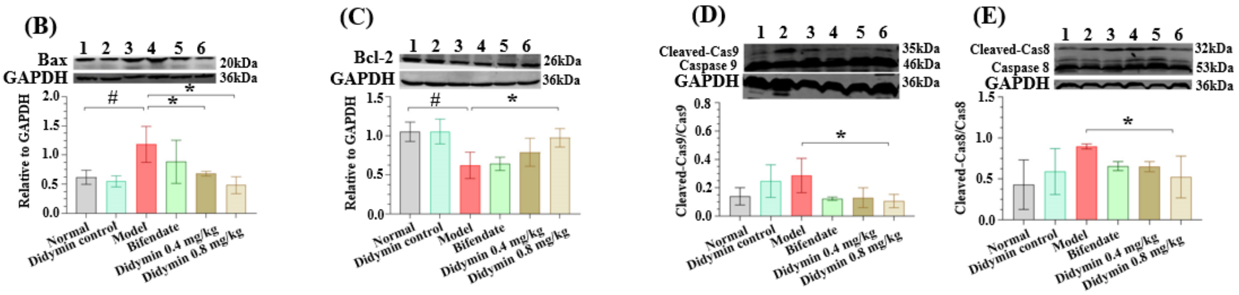

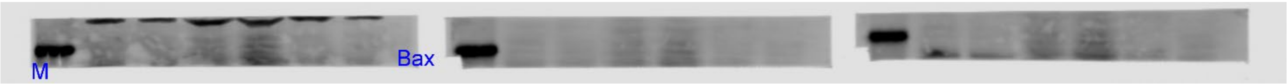

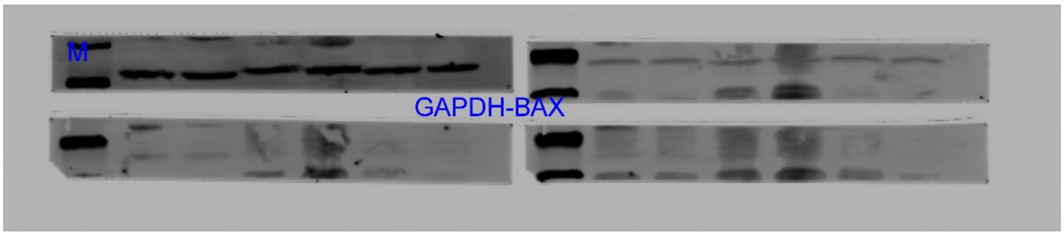

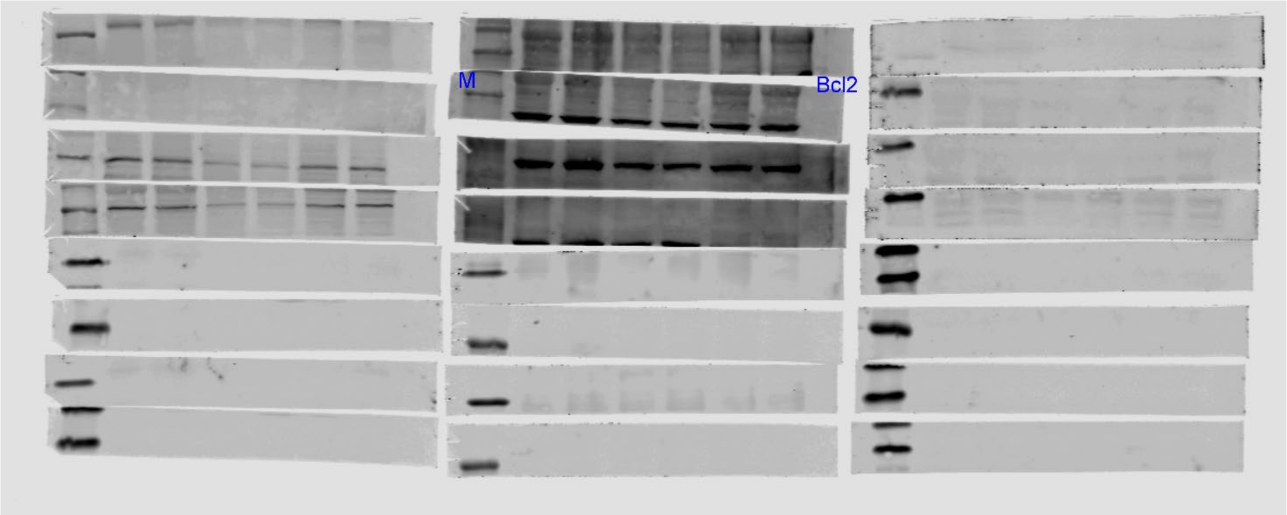
M
Bcl2

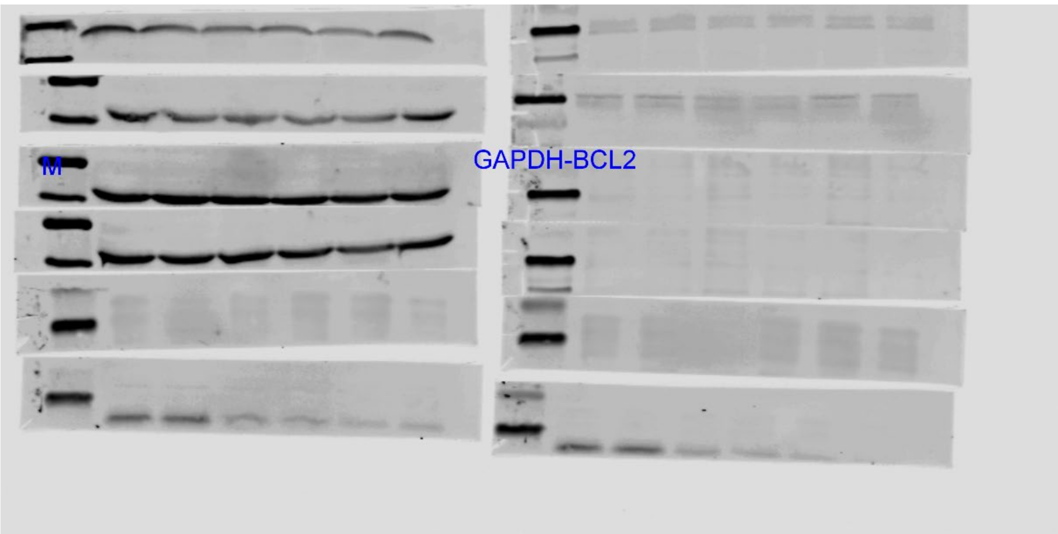
M
GAPDH-BCL2

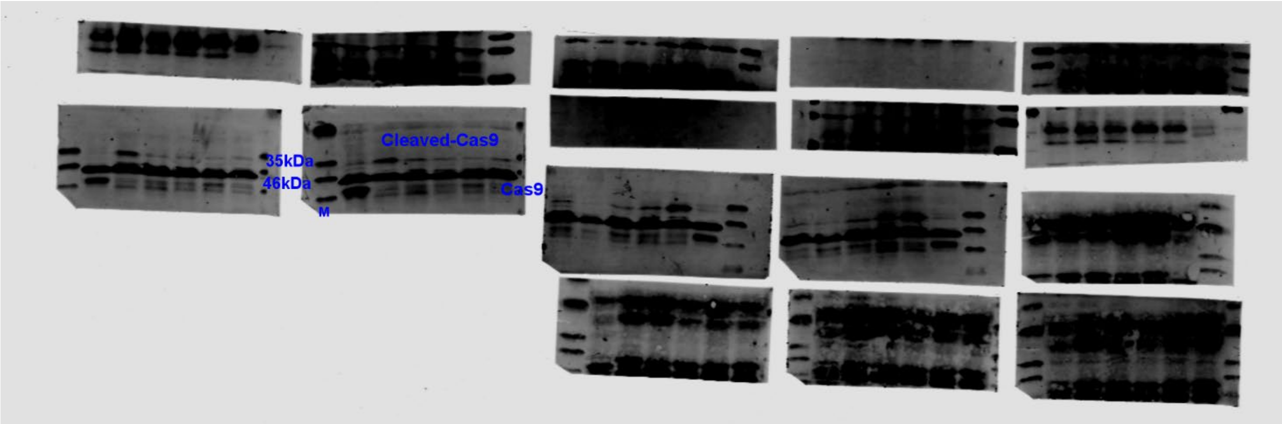
35kDa
46kDa
M
Cleaved-Cas9
Cas9

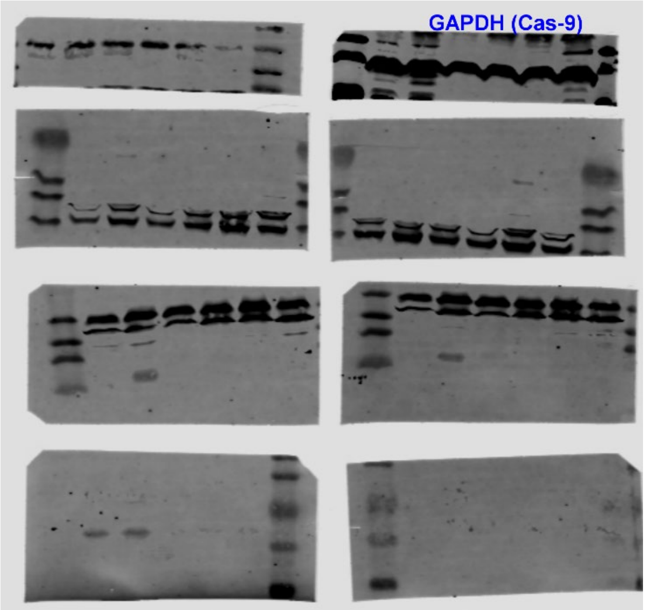
GAPDH (Cas-9)

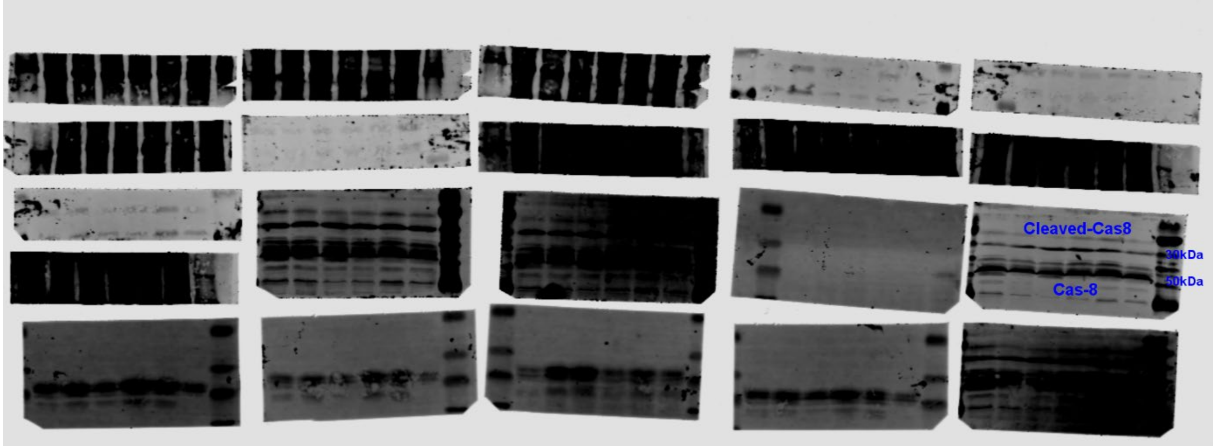
Cleaved-Cas8
30kDa
50kDa
Cas-8

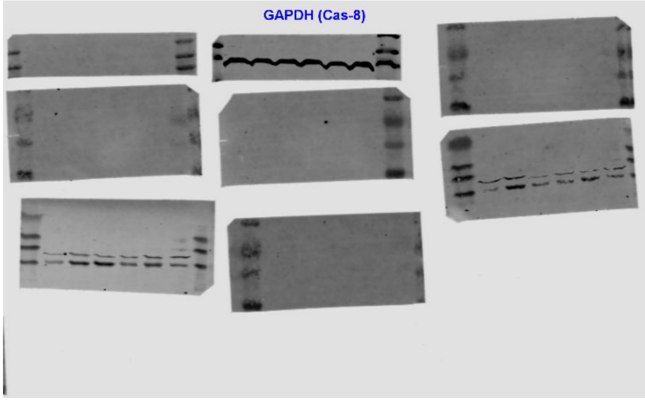
GAPDH (Cas-8)

Figure 6 p-PI3K/PI3K, p-Akt/Akt, p-mTOR/mTOR, P70S6K and PTEN

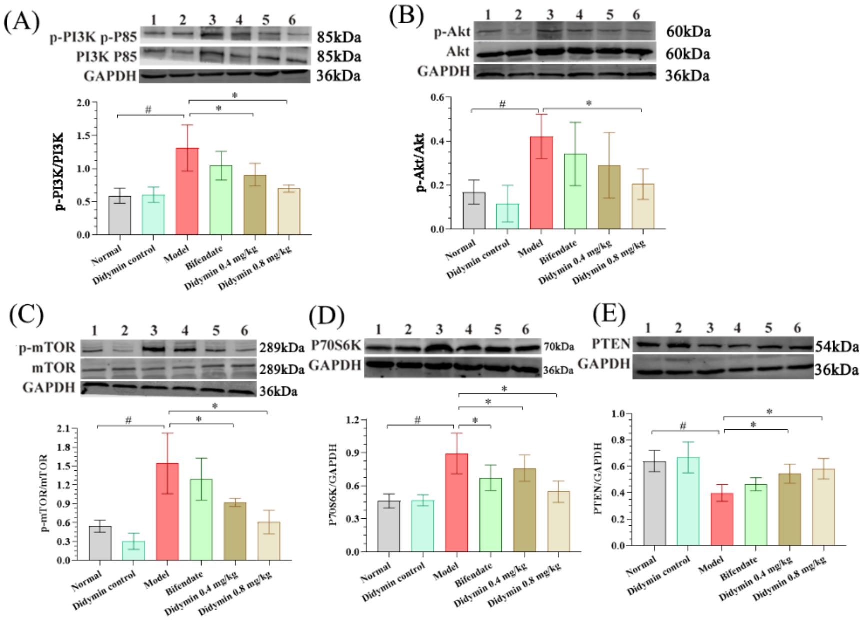

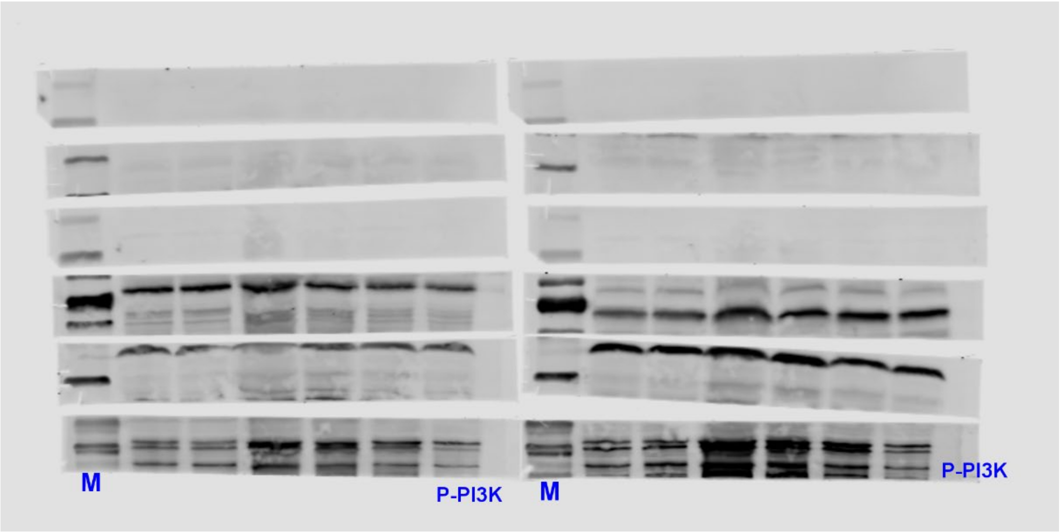

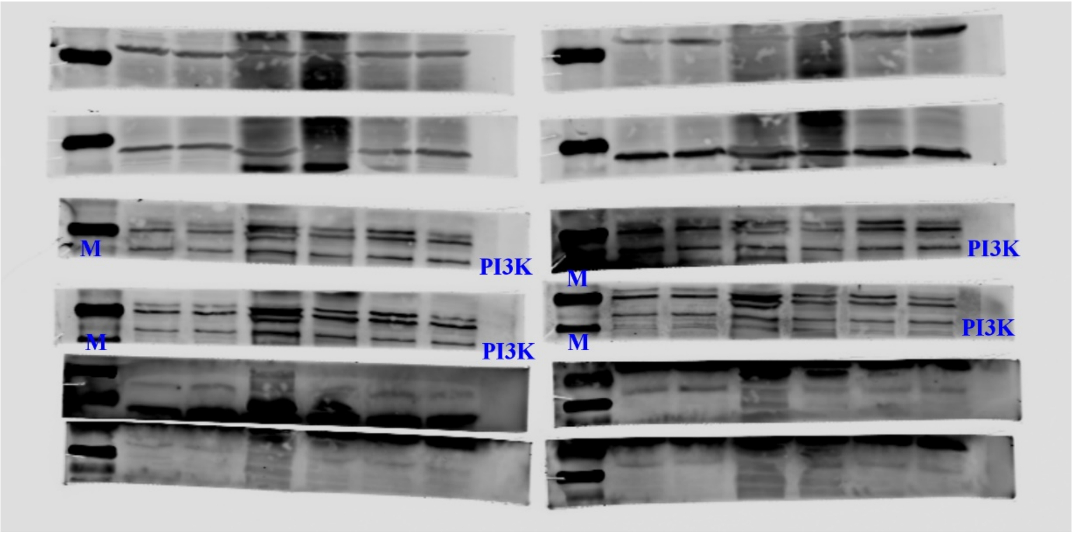

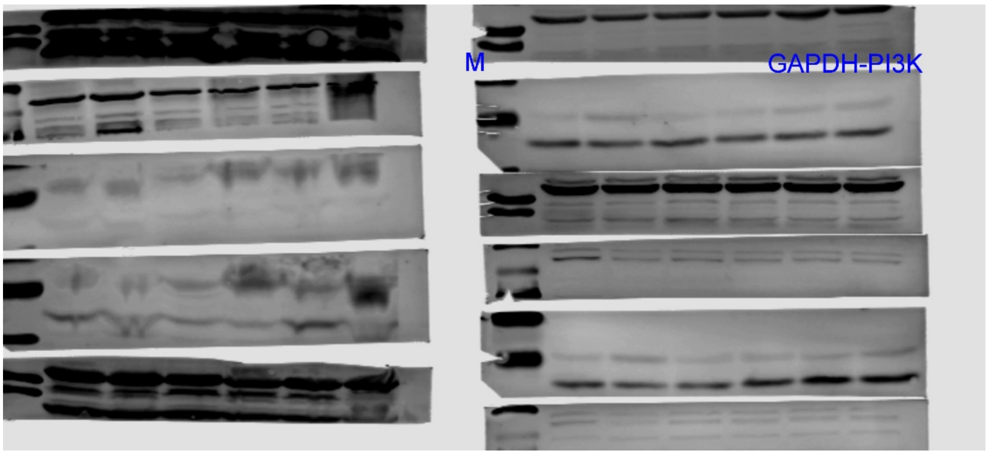
M
GAPDH-PI3K

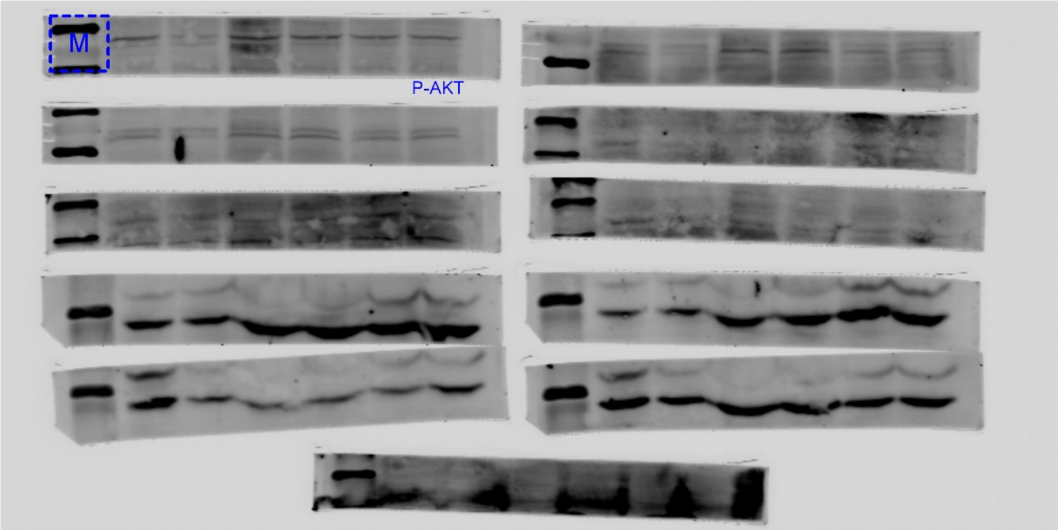
M
P-AKT

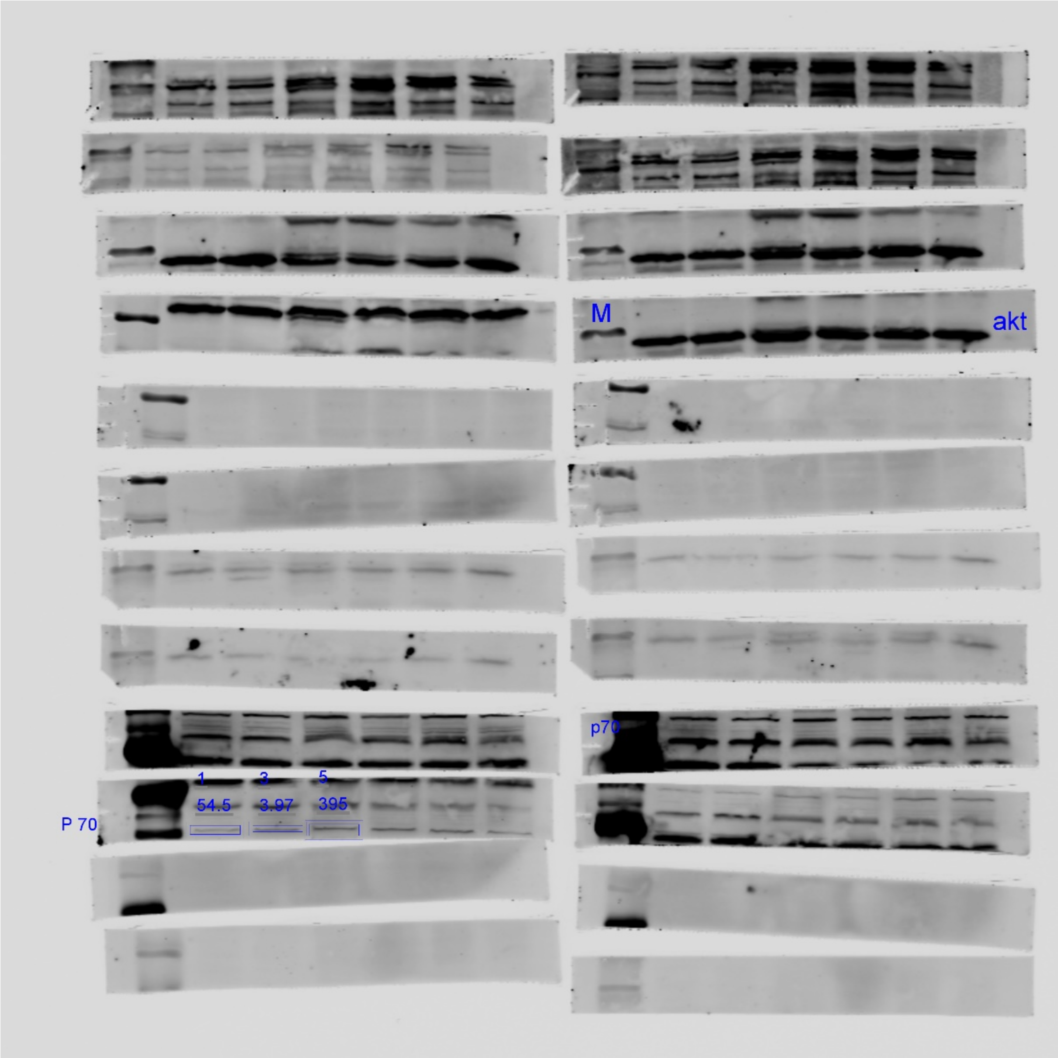
M
akt
p70
1
3
5
54.5
3.97
395
P 70

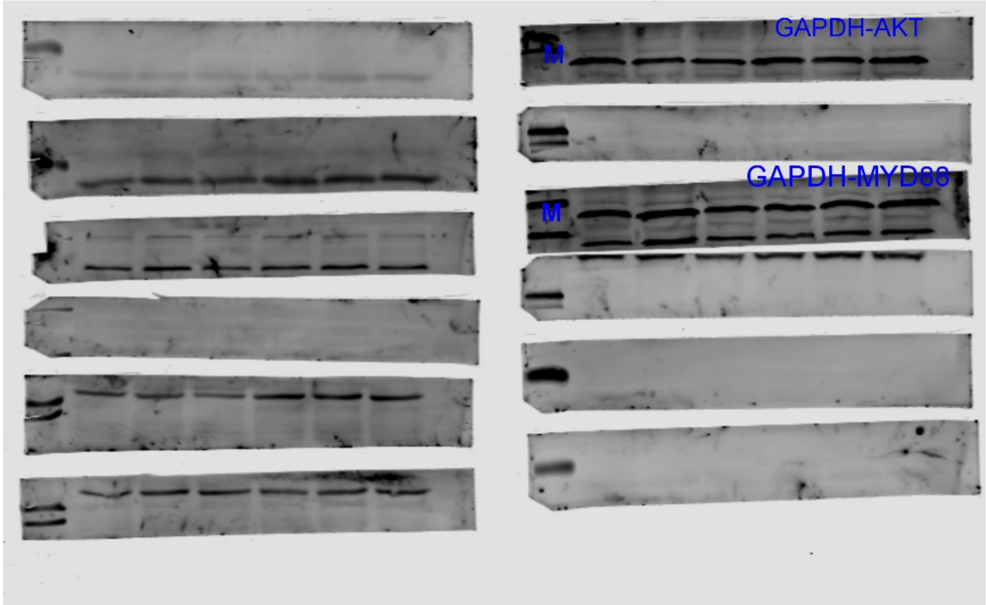
GAPDH-AKT
M
GAPDH-MYD88
M

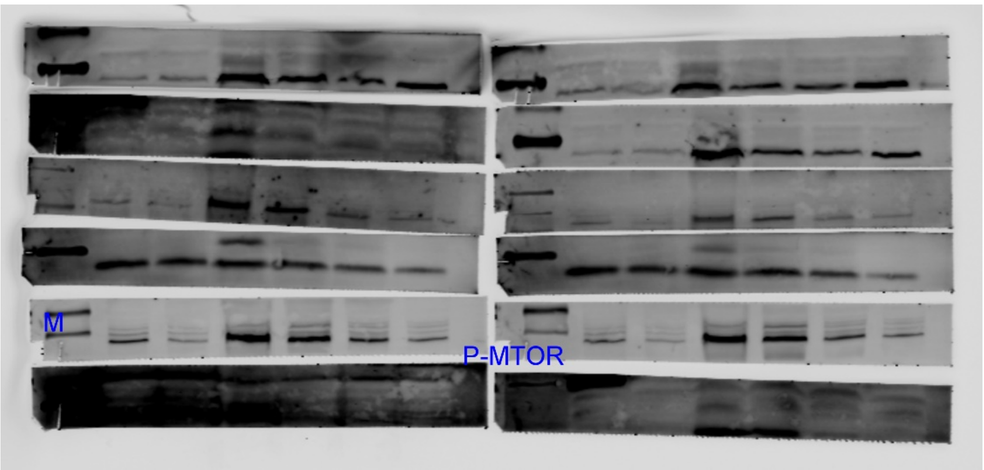
M
P-MTOR

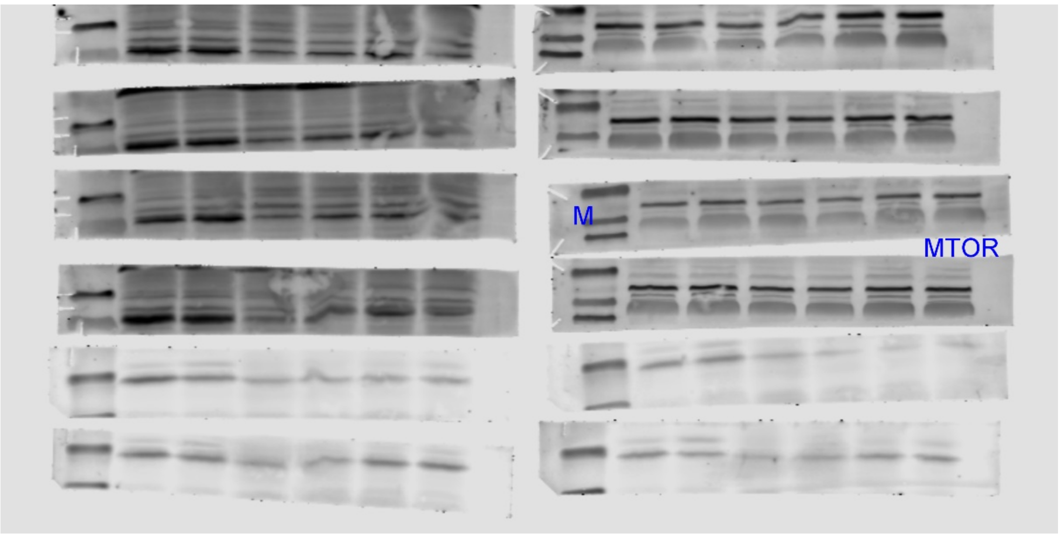
M
MTOR

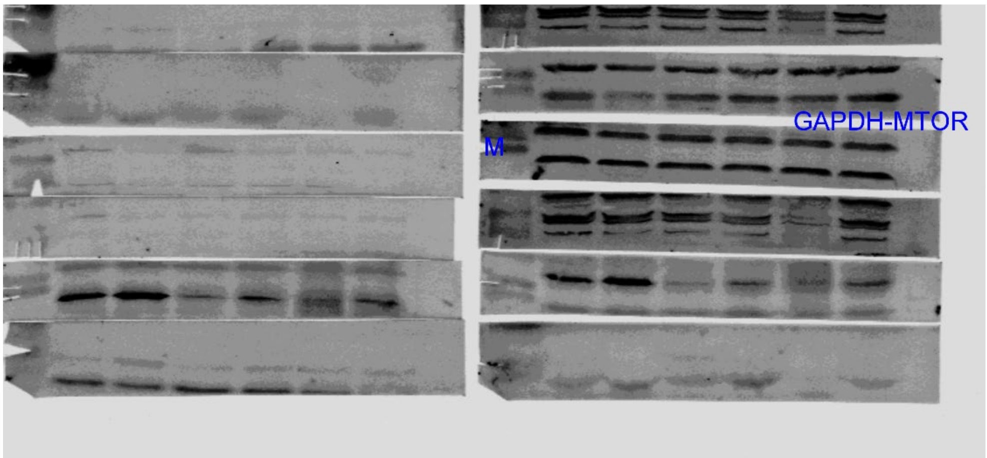
GAPDH-MTOR
M

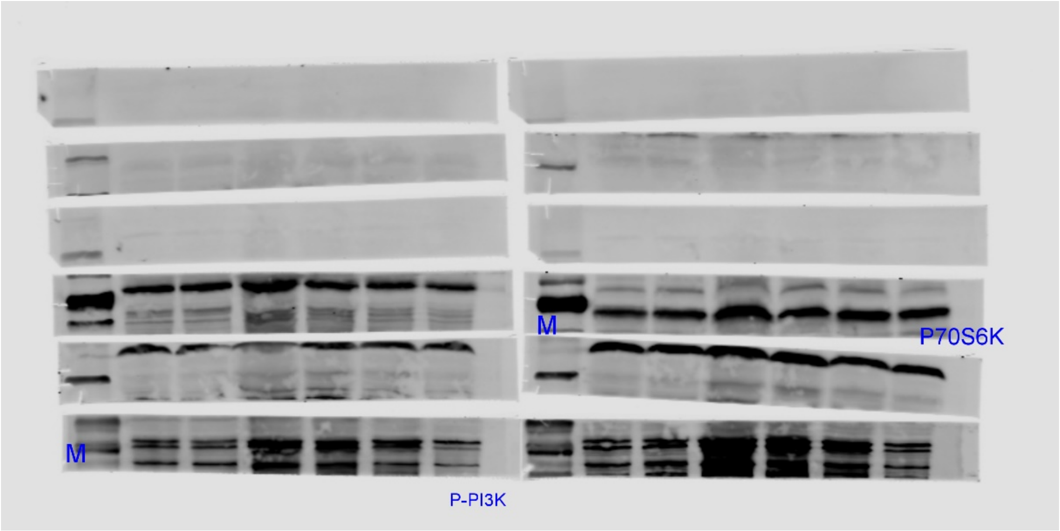
M
P70S6K
M
P-PI3K

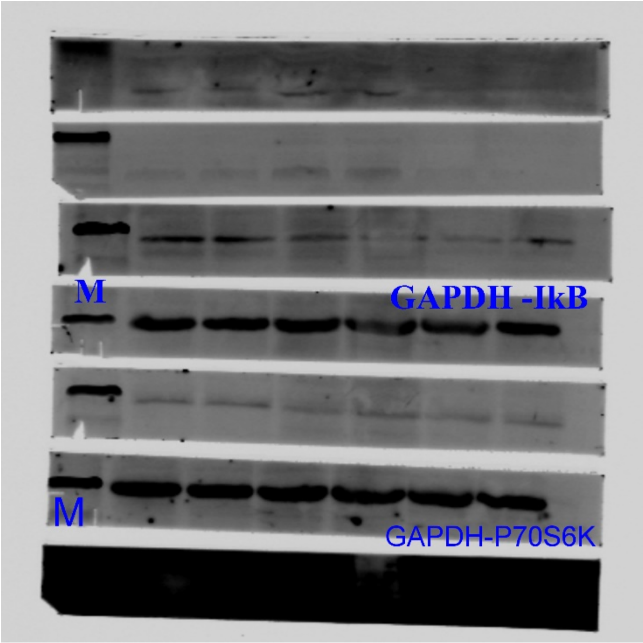
M
GAPDH -IkB
M
GAPDH-P70S6K

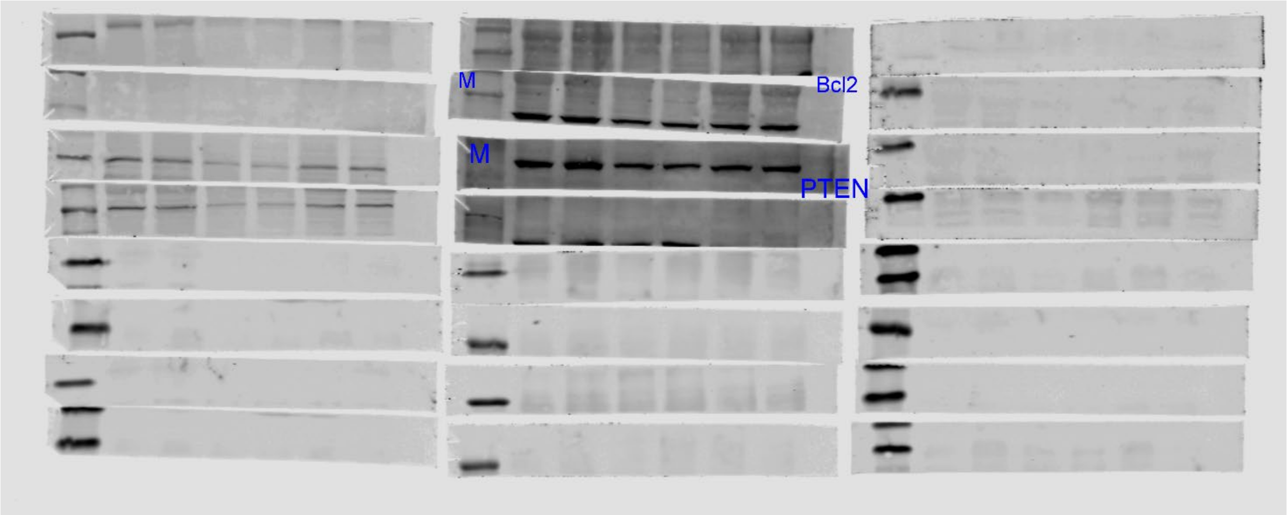
M
Bcl2
M
PTEN

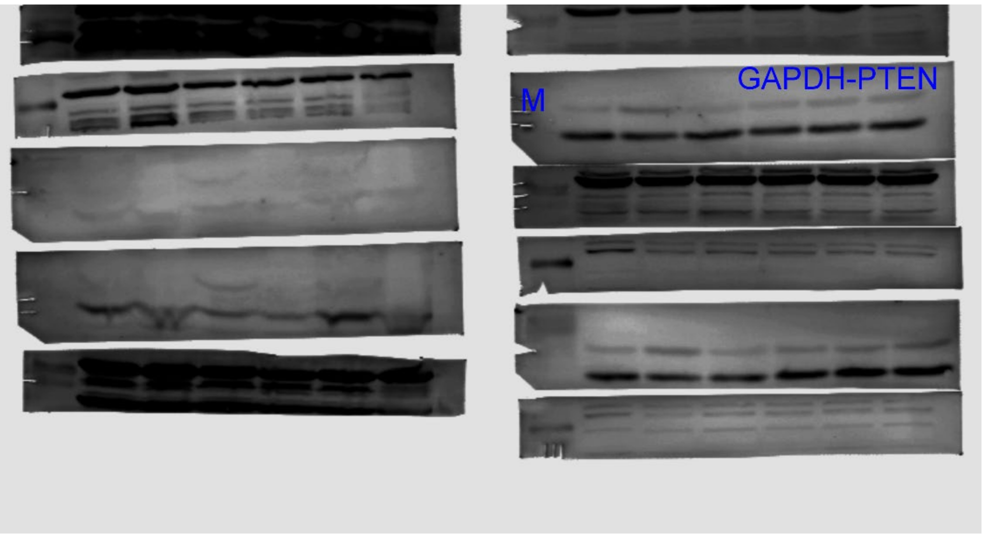

Fig. 7 Didymin inhibited the TLR4/NF-κB pathway. (A) The immunohistochemical analysis of NF-κB

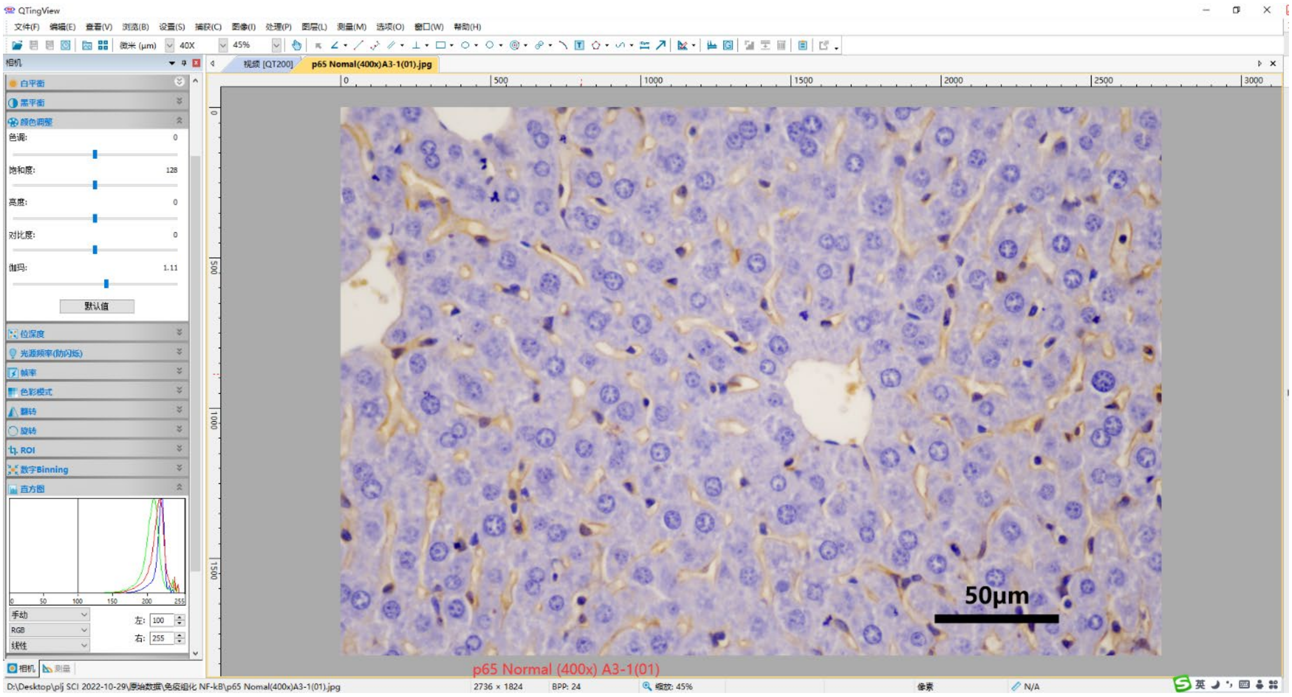

Normal group

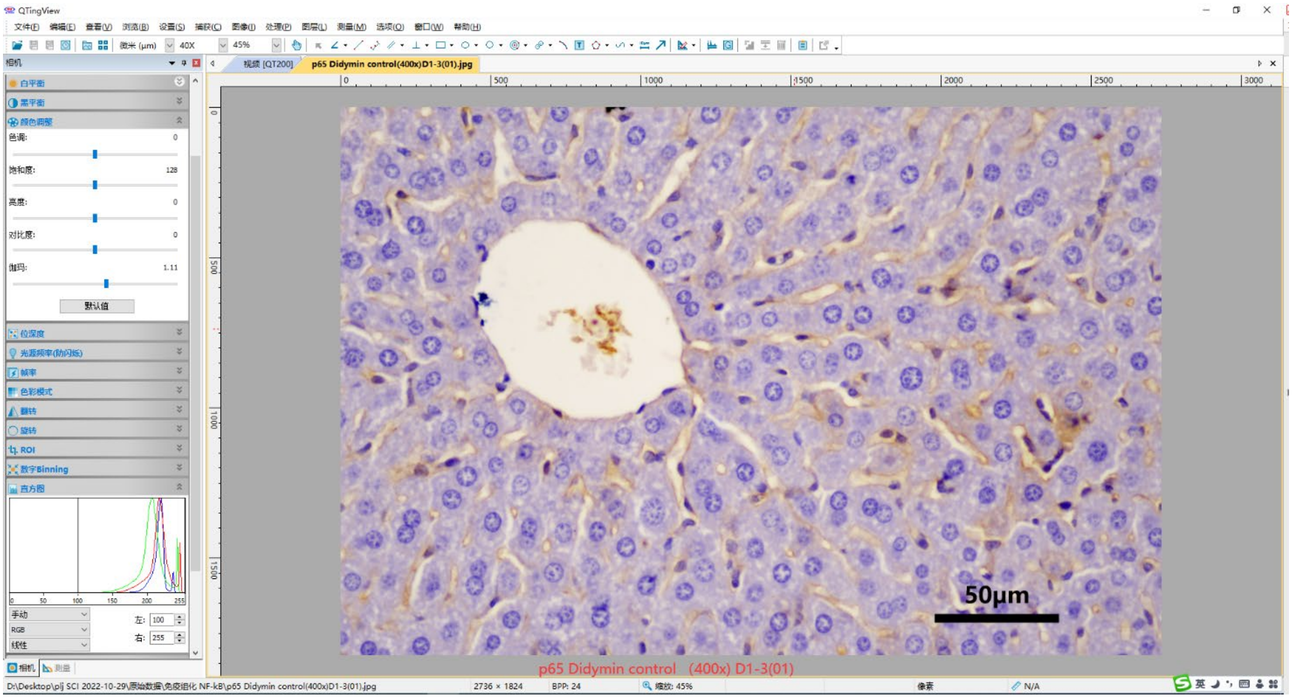

Didymin control group

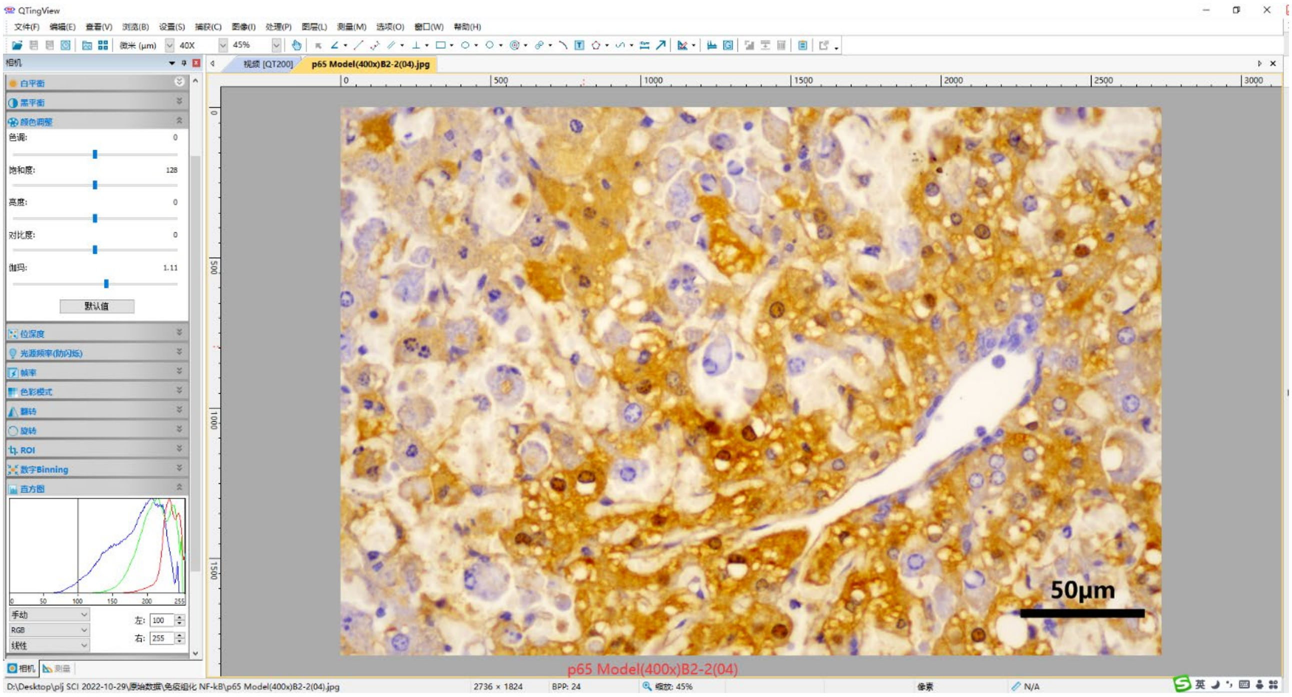

Model group

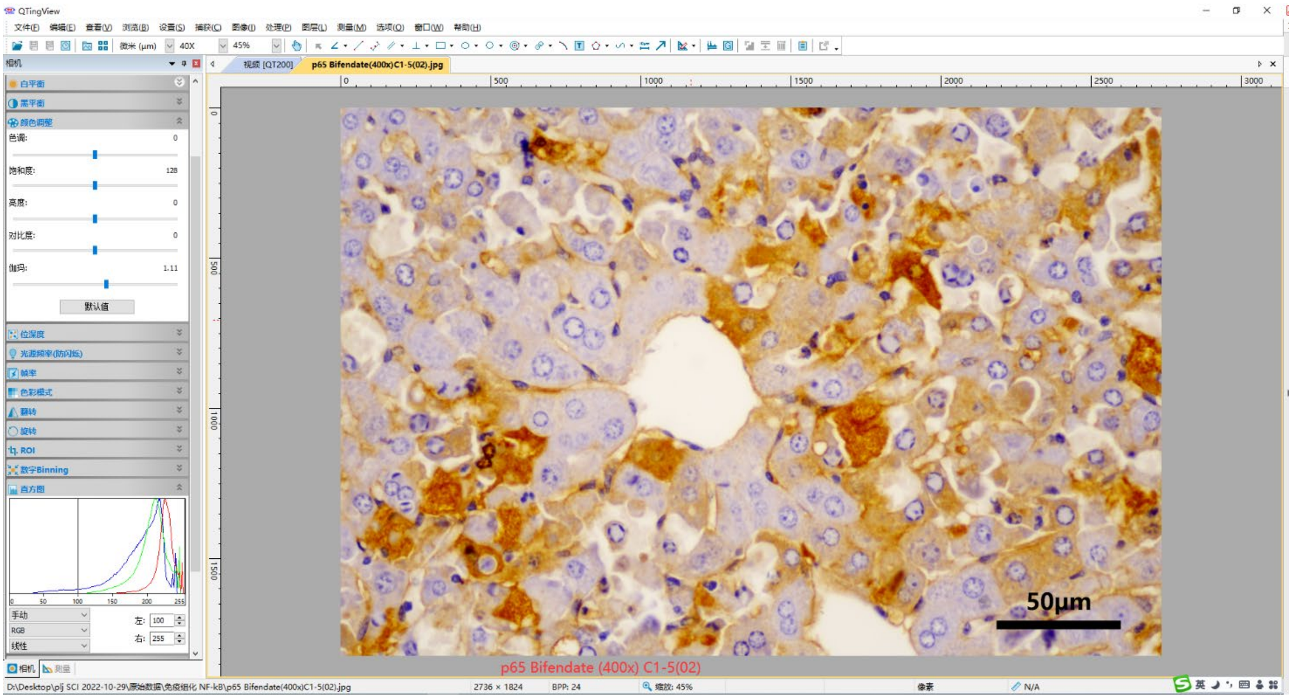

Bifendate group

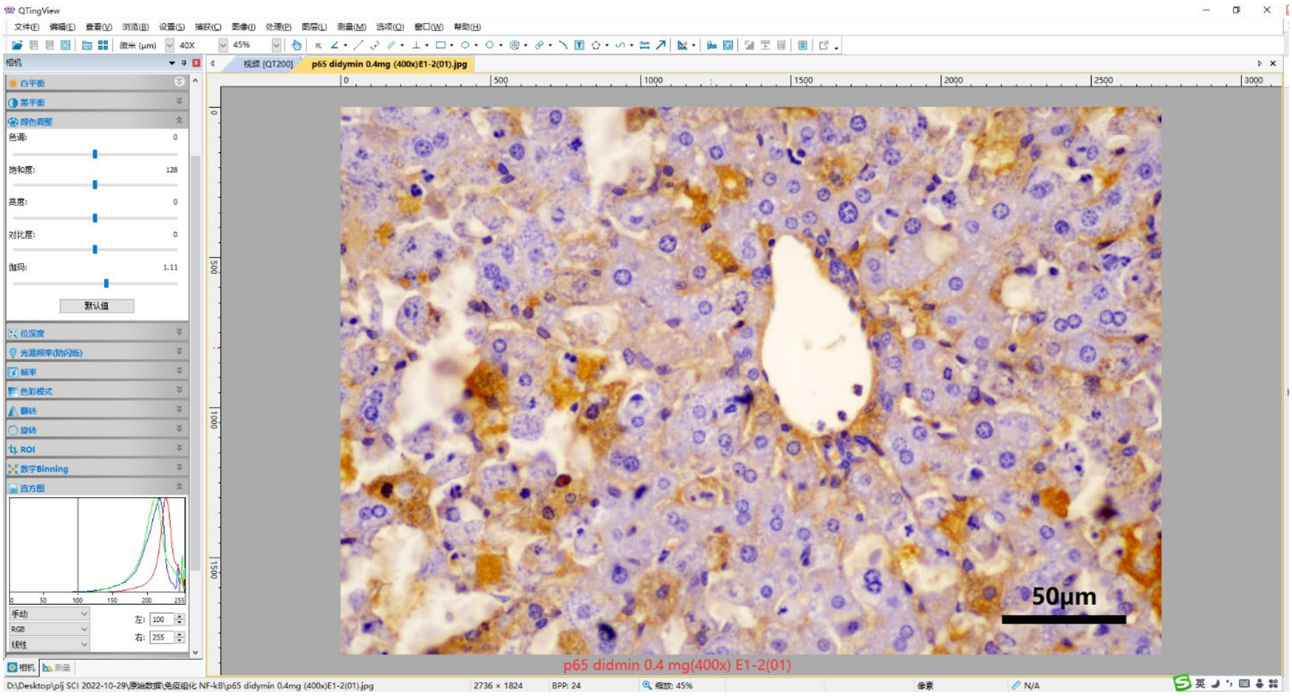

Didymin-treated group (0.4 mg/kg)

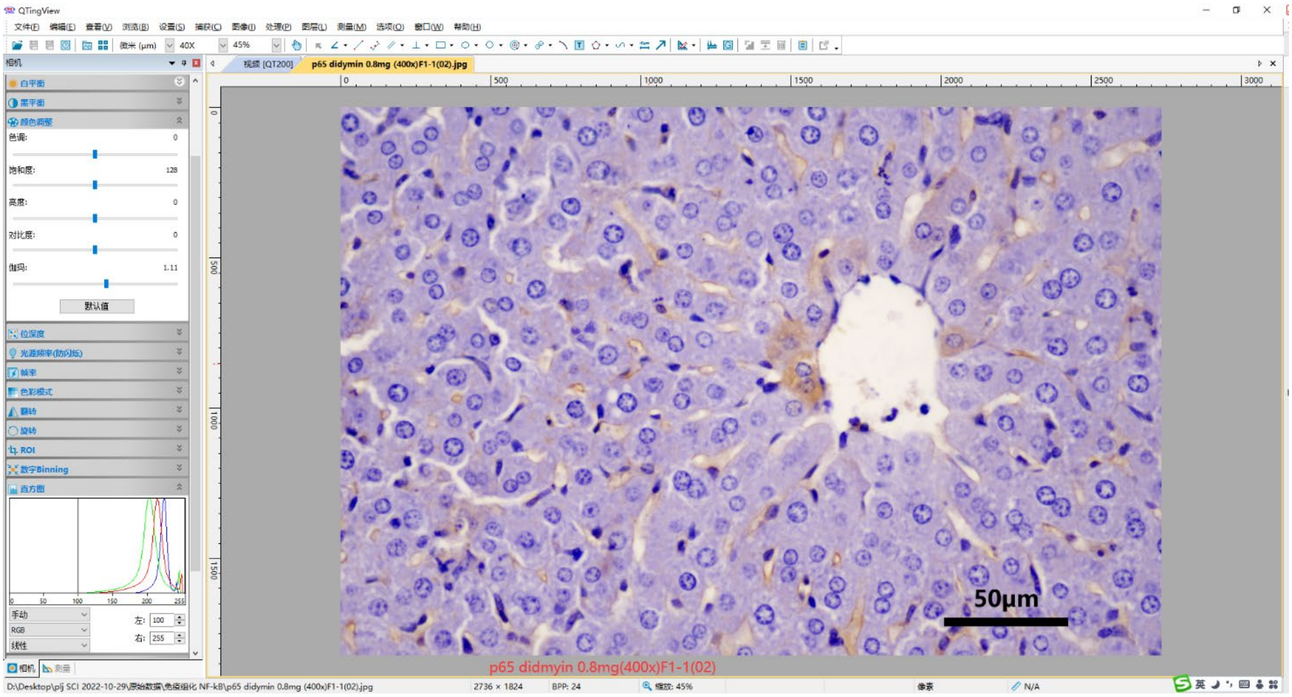

Didymin-treated group (0.8 mg/kg)

Figure 7 TLR4, MyD88, NF-κB, p-IKKα/β/IKKα/β and p-IκBα/IκBα

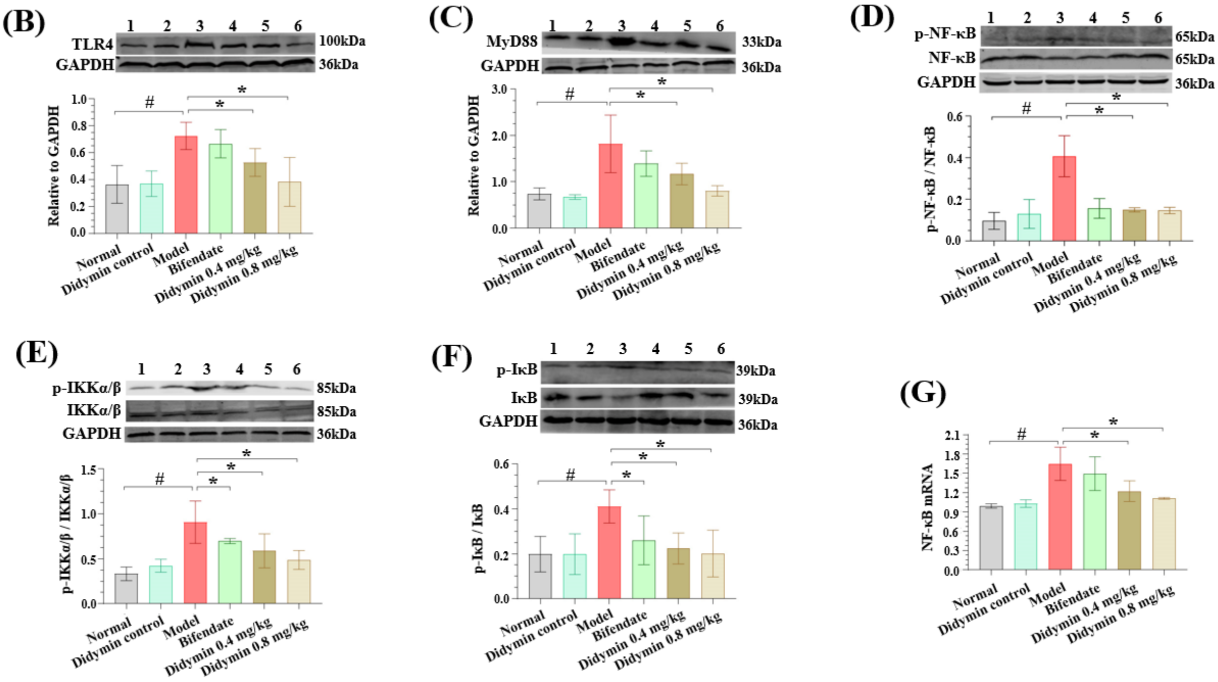

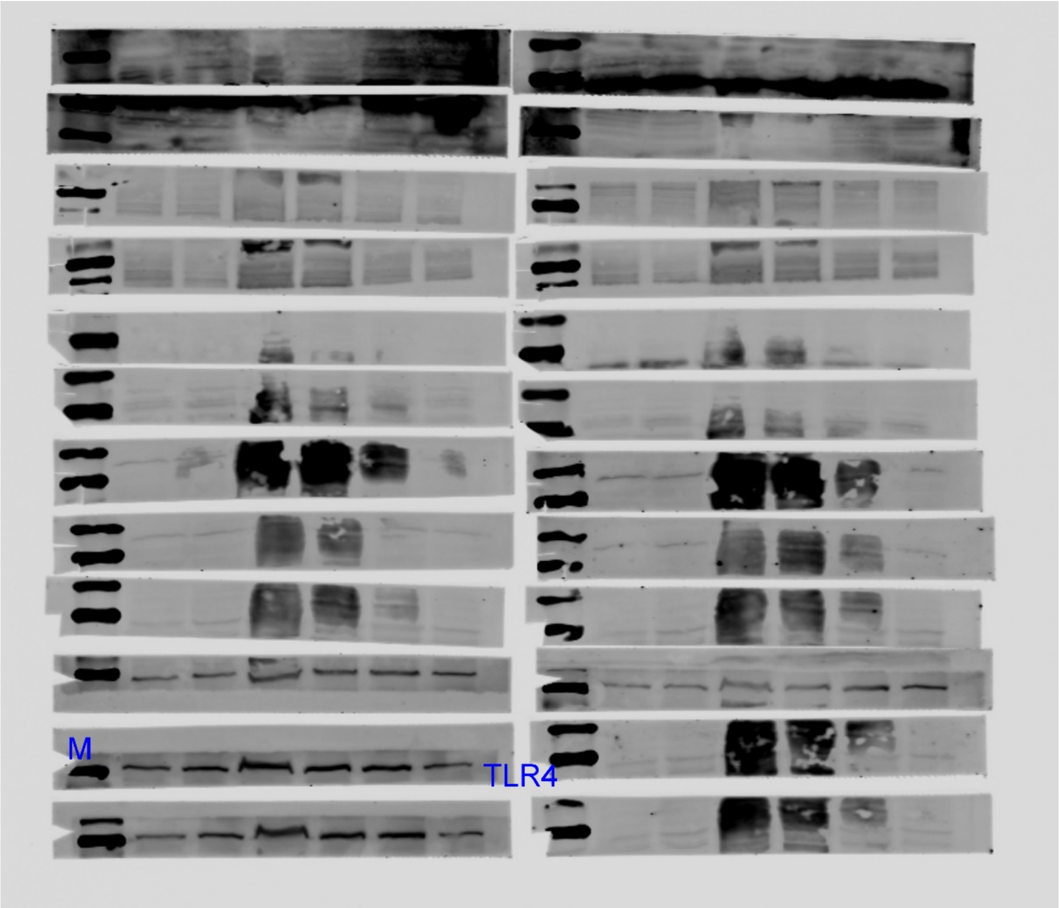
M
TLR4

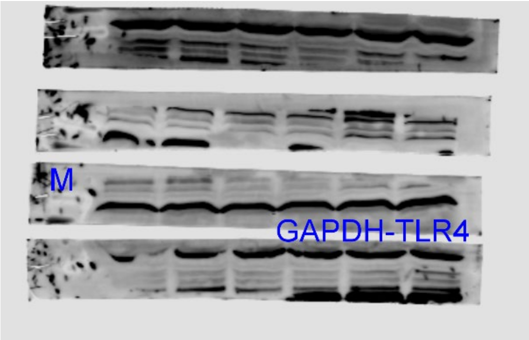
M
GAPDH-TLR4

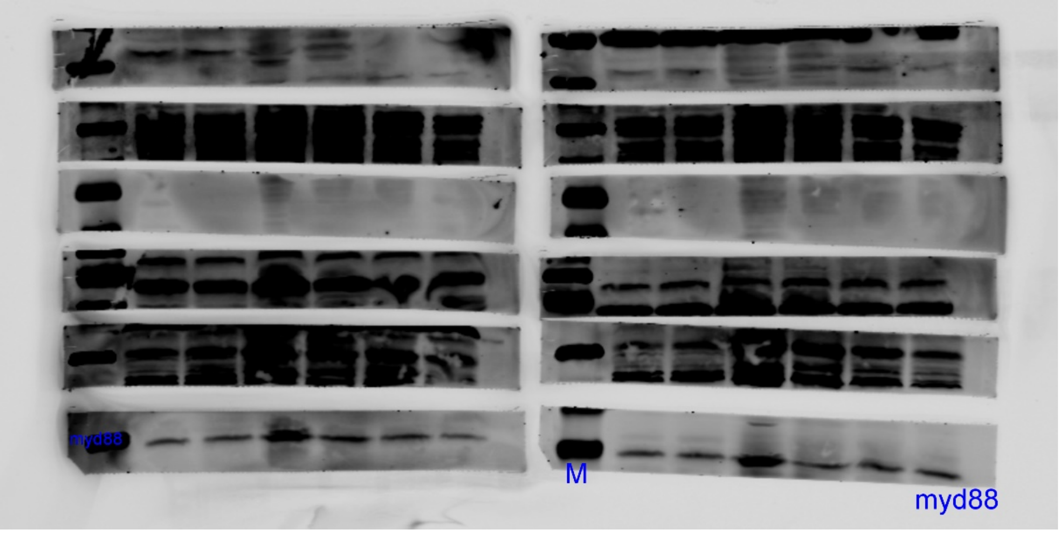
M
myd88

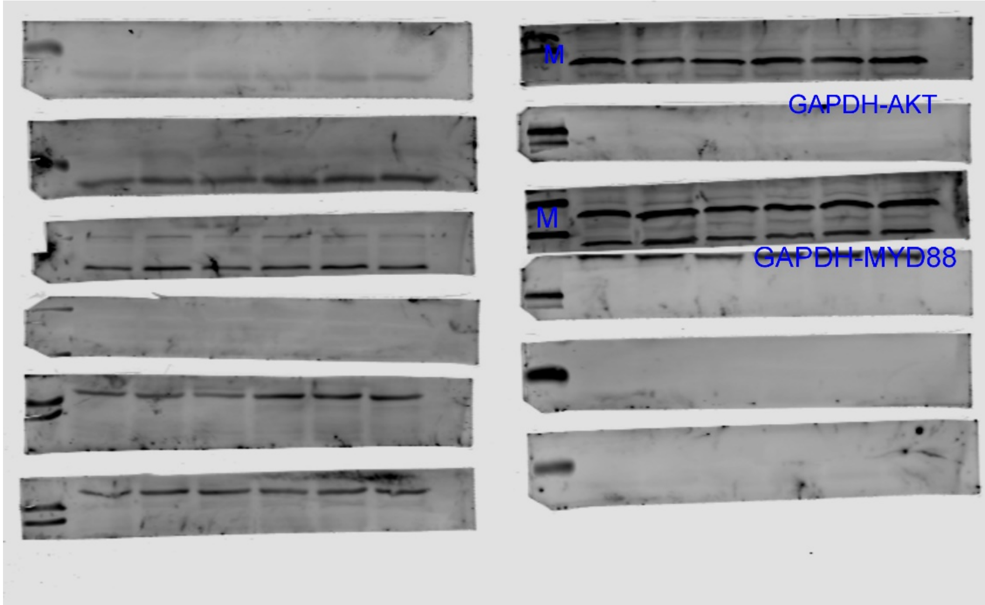
M
GAPDH-AKT
M
GAPDH-MYD88

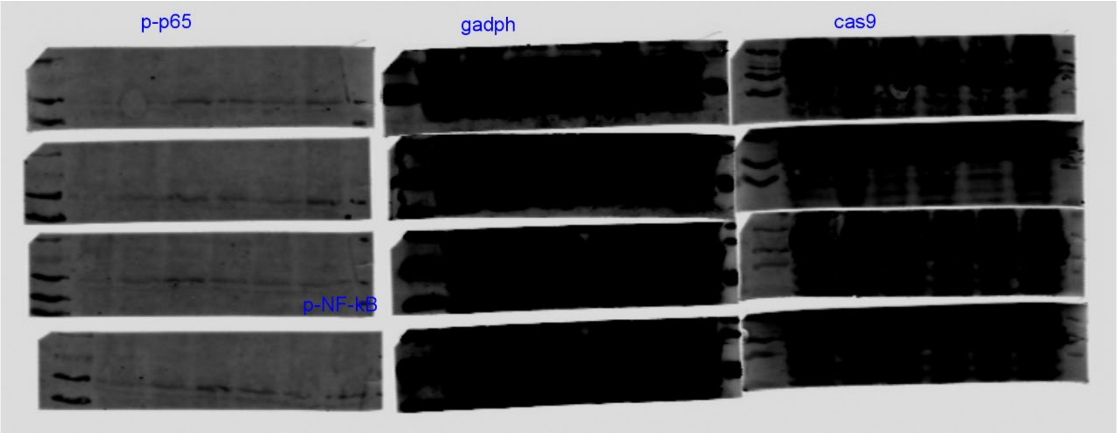
p-p65
gadph
cas9
p-NF-kB

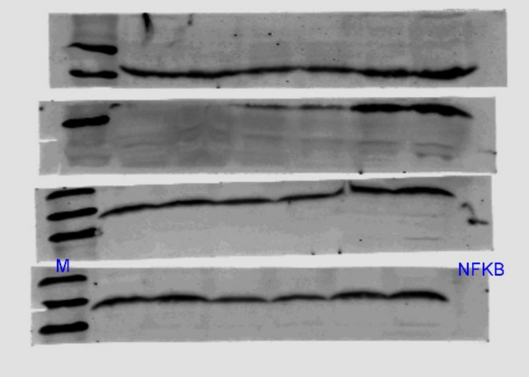
M
NFKB

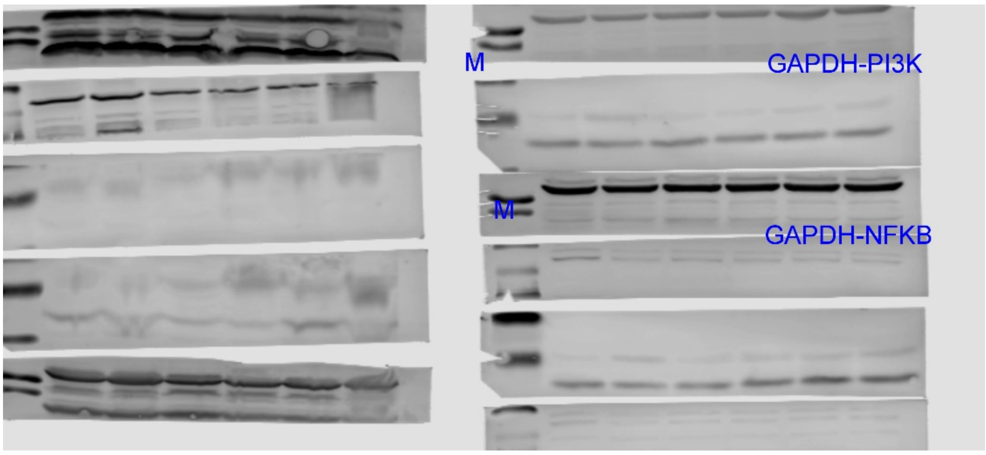
M
GAPDH-PI3K
M
GAPDH-NFKB

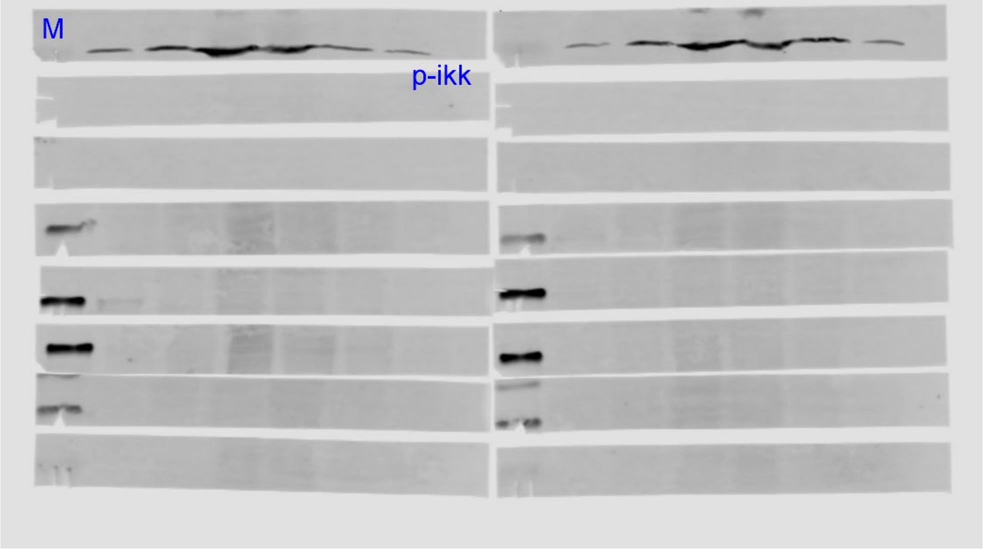
M
p-ikk

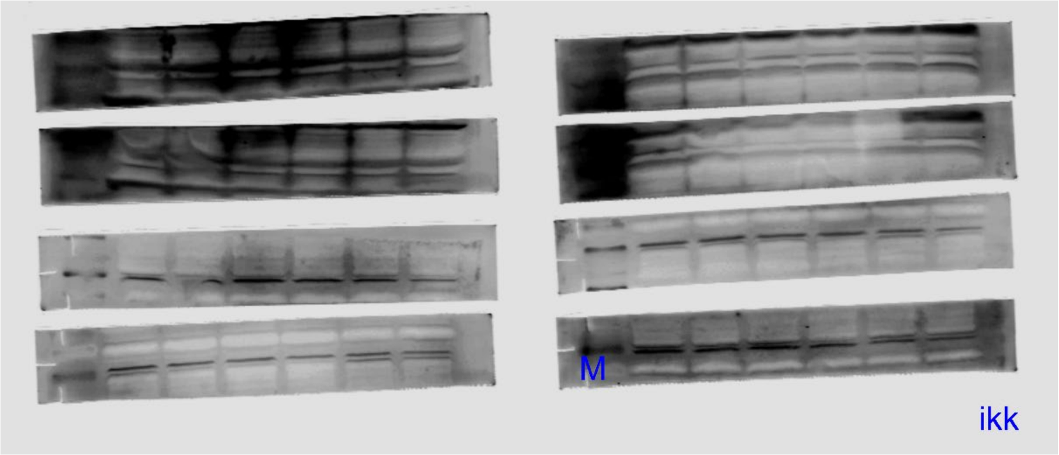
M
ikk

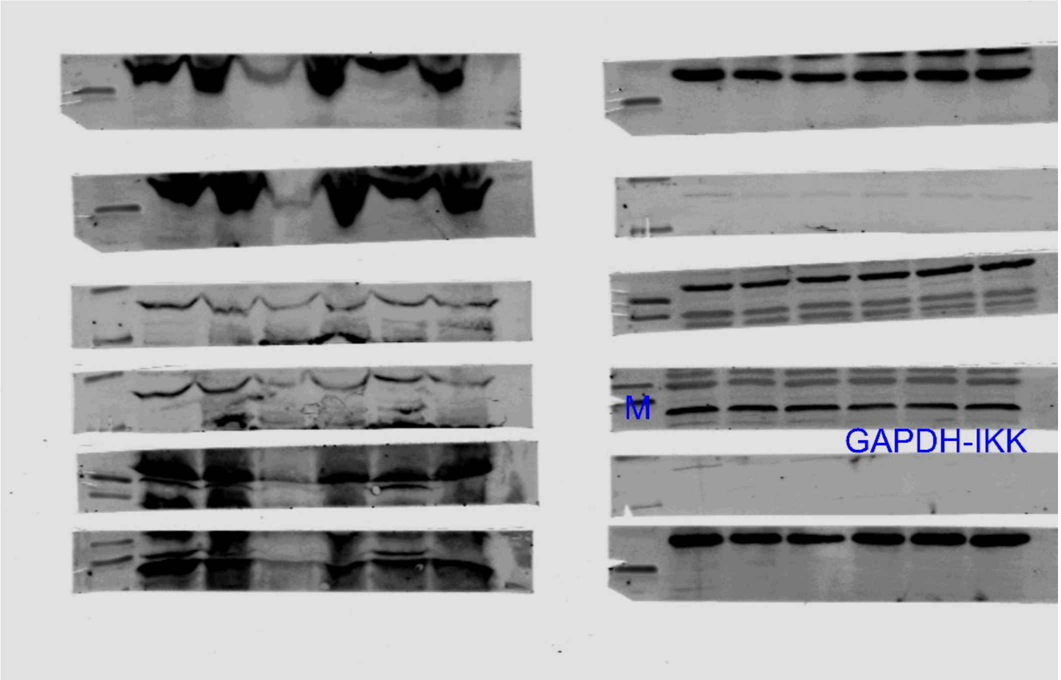
M
GAPDH-IKK

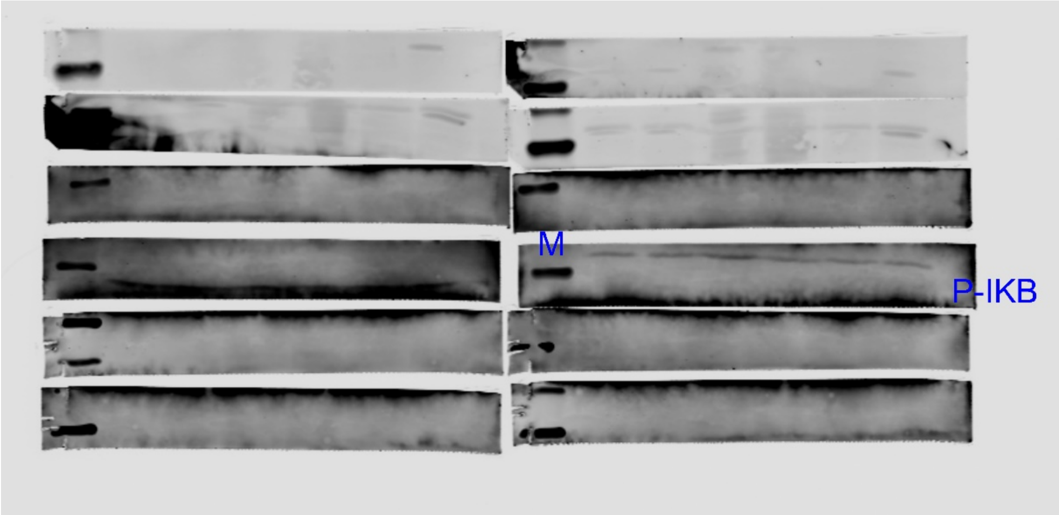
M
P-IKB

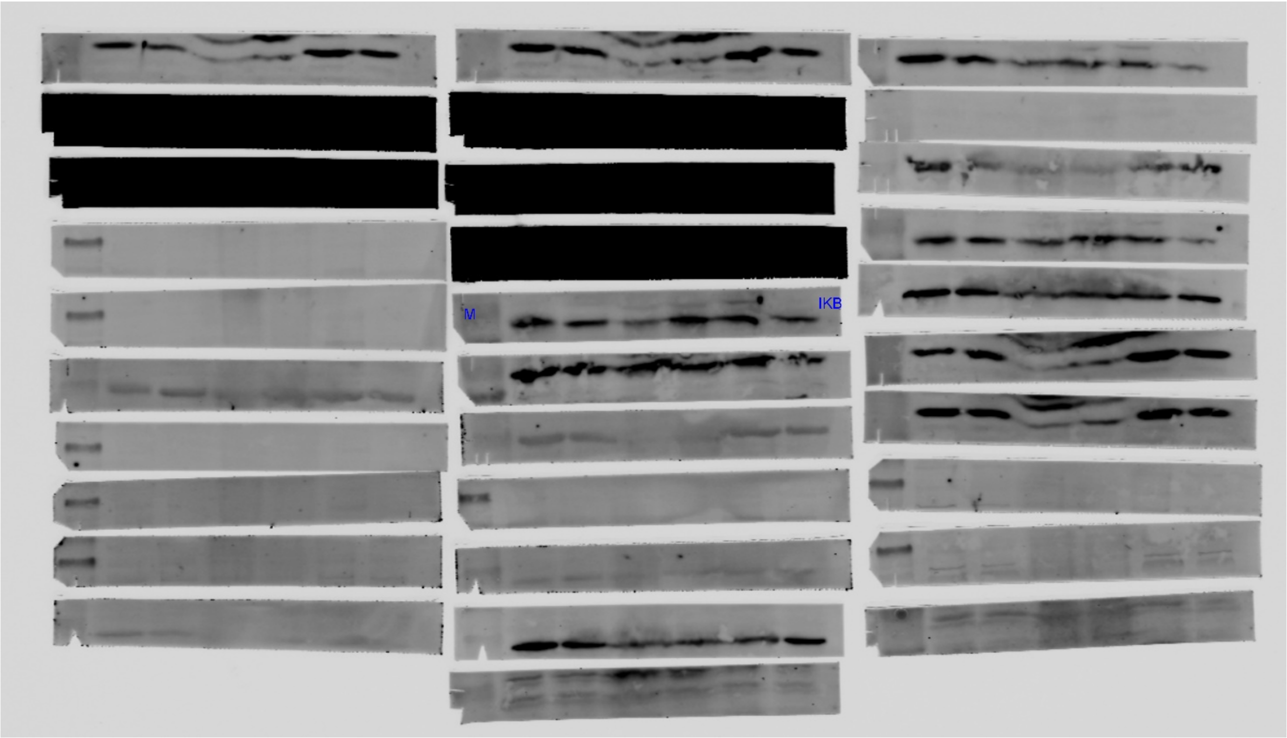
M
IKB

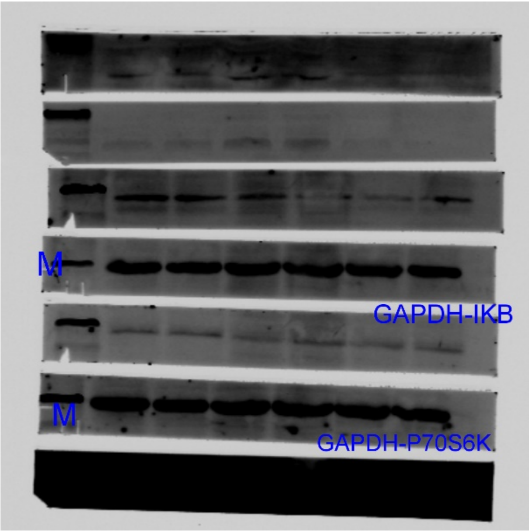
M
GAPDH-IKB
M
GAPDH-P70S6K
